# An independent regulator of global release pathways in astrocytes generates a subtype of extracellular vesicles required for postsynaptic function

**DOI:** 10.1126/sciadv.adg2067

**Published:** 2023-06-23

**Authors:** Reuben Levy-Myers, Daniel Daudelin, Chan Hyun Na, Shanthini Sockanathan

**Affiliations:** ^1^ The Solomon Snyder Department of Neuroscience, Johns Hopkins University School of Medicine, PCTB1004, 725 N. Wolfe Street, Baltimore, MD 21205, USA.; ^2^ Department of Neurology, Institute for Cell Engineering, Johns Hopkins University School of Medicine, MRB 706, 733 N. Broadway, Baltimore, MD 21205, USA.

## Abstract

Extracellular vesicles (EVs) are heterogeneous in size, composition, and function. We show that the six-transmembrane protein glycerophosphodiester phosphodiesterase 3 (GDE3) regulates actin remodeling, a global EV biogenic pathway, to release an EV subtype with distinct functions. GDE3 is necessary and sufficient for releasing EVs containing annexin A1 and GDE3 from the plasma membrane via Wiskott-Aldrich syndrome protein family member 3 (WAVE3), a major regulator of actin dynamics. GDE3 is expressed in astrocytes but not neurons, yet mice lacking GDE3 [*Gde3* knockout (KO)] have decreased miniature excitatory postsynaptic current (mEPSC) amplitudes in hippocampal CA1 neurons. EVs from cultured wild-type astrocytes restore mEPSC amplitudes in *Gde3* KOs, while EVs from *Gde3* KO astrocytes or astrocytes inhibited for WAVE3 actin branching activity do not. Thus, GDE3-WAVE3 is a nonredundant astrocytic pathway that remodels actin to release a functionally distinct EV subtype, supporting the concept that independent regulation of global EV release pathways differentially regulates EV signaling within the cellular EV landscape.

## Teaser

GDE3 regulates actin dynamics via WAVE3 to release a subtype of EVs from astrocytes important for postsynaptic activity.

## INTRODUCTION

Extracellular vesicles (EVs) are small membranous vesicles released by all cells that are highly heterogeneous in size and composition. They carry proteins, lipids, and nucleic acids that can be taken up by recipient cells, allowing for complex signals to be delivered in a targeted fashion (*1*). Accordingly, EVs are now considered important conduits of intercellular communication. EVs vary greatly in terms of physical properties, cargo, and signaling output. How this heterogeneity is regulated remains unclear. This diversity is not simply a stochastic process, as cells can tune EV output to control the functional consequence of the EVs that they produce. For example, in the nervous system, astrocytes can release EVs that affect neuronal activity in unique ways. EVs produced by astrocytes treated with interleukin-1β increase the frequency of neuronal firing, while EVs produced by adenosine 5´-triphosphate (ATP)–stimulated astrocytes have the opposite effect of reducing firing frequency (*2*). These observations suggest that there are cellular mechanisms in place that can regulate the release of EVs with distinct functional properties in a context-dependent manner. Systemic stimulus-dependent changes in the cellular transcriptome or proteome may contribute to the generation of functionally distinct EVs; however, the observation that EV cargoes can diverge considerably from cellular abundance suggests that other mechanisms are in play (*3*–*6*). One possibility is that the release of functionally distinct EV subtypes is controlled by designated cellular pathways and that these pathways comprise an important regulatory node that determines EV subtype production. Deeper insight into how EVs are released will help clarify this question.

EVs are stratified into two major classes according to their release modes (*1*). Exosomes are produced by inward budding of the membrane of a multivesicular body (MVB) and are released from the cell after MVB fusion with the plasma membrane, while microvesicles (MVs) are produced by direct outward budding of the plasma membrane. These different production routes result in exosomes and MVs having divergent cargos, regulation, and release kinetics. Global exosome biogenesis pathways involve the ESCRT (endosomal sorting complex required for transport) pathway (*7*), ceramide remodeling via neutral sphingomyelinase 2 activity (*8*), and tetraspanin proteins (CD63/CD81/CD9) (*1*). The production of MVs is more varied and less well-understood. Some MVs are released by mechanisms shared by exosomes such as ESCRTs and sphingomyelinases, whereas other MVs are generated by separate biogenic machinery that includes actin remodeling (*1*, *7*), ARF6 (adenosine 5´-diphosphate ribosylation factor 6) (*9*), and specialized pathways present in specific cell types or contexts such as cancer (*10*). EVs are extremely heterogeneous, yet there are comparatively few biogenic pathways suggesting that there is not a one-to-one ratio where each EV subtype is produced by one dedicated biogenic route. Instead, cells may be able to modulate these broad pathways in specific ways to release functionally distinct EV subtypes. Whether and how known exosome and MV biogenesis pathways contribute to controlling the heterogeneity of EVs are still unclear.

Astrocytes provide an excellent model to examine the mechanisms by which different subtypes of EVs are released. Astrocytes are known to signal to neurons in a multifaceted way that includes the release and uptake of small molecules, metabolites, soluble proteins, and, more recently, exosomes and MVs (*11*, *12*). For example, astrocytes can release EVs that are neuroprotective and play roles in neurogenesis (*11*); moreover, they can respond to stimuli such as anti-inflammatory cytokines by releasing EVs that modulate neuronal plasticity, neurite extension, and survival (*2*, *6*). These observations indicate that astrocytes can dynamically regulate the release of functionally distinct EVs under different conditions, suggesting that regulated EV release is an important feature of astrocyte function throughout life. Accordingly, studies in astrocytes are likely to shed light on how cells control EV production to produce complex and varied physiological responses.

Glycerophosphodiester phosphodiesterase 3 (GDE3, *Gdpd2*) is a six-transmembrane protein that is expressed primarily in astrocytes and, to a lesser extent, in oligodendrocyte precursor cells (OPCs) in the developing spinal cord (*13*). GDE3 contains an enzymatic domain that faces the extracellular space (*13*), which can cleave the glycosylphosphatidylinositol (GPI) anchor that tethers some proteins to the membrane. Cleavage of select GPI–anchored protein (GPI-AP) substrates by GDE3 inhibits cellular proliferation cells autonomously in OPCs and cancer cells (*13*, *14*). GDE3 can also metabolize lysophosphatidylinositol (LPI) lipids which may modulate endocannabinoid signaling (*15*, *16*). In addition to these functions, GDE3 was recently found to increase the release of the GPI-AP CNTFRα (ciliary neurotrophic factor receptor subunit alpha) in EVs through mechanisms independent of its enzymatic function (*13*); however, broader roles for GDE3 in EV biology have not been tested.

Here, we show that GDE3 strongly induces the release of a specific subtype of MV from the plasma membrane that is molecularly defined by the presence of annexin A1 and GDE3 itself. GDE3 releases these MVs by remodeling the actin cytoskeleton via the WAVE regulatory complex (WRC). In the postnatal hippocampus, GDE3 is primarily expressed in astrocytes, where it is required to release EVs that are required for appropriate neuronal postsynaptic function. Thus, GDE3 regulation of WAVE3 integrates with actin-dependent pathways of MV release to generate a subset of EVs that are important for synaptic function, supporting the concept that EV functional selectivity is governed, in part, by the controlled release of EV subtypes via distinct regulatory pathways that feed into global EV release mechanisms.

## RESULTS

### GDE3 induces the release of MVs

**Fig. 1.**
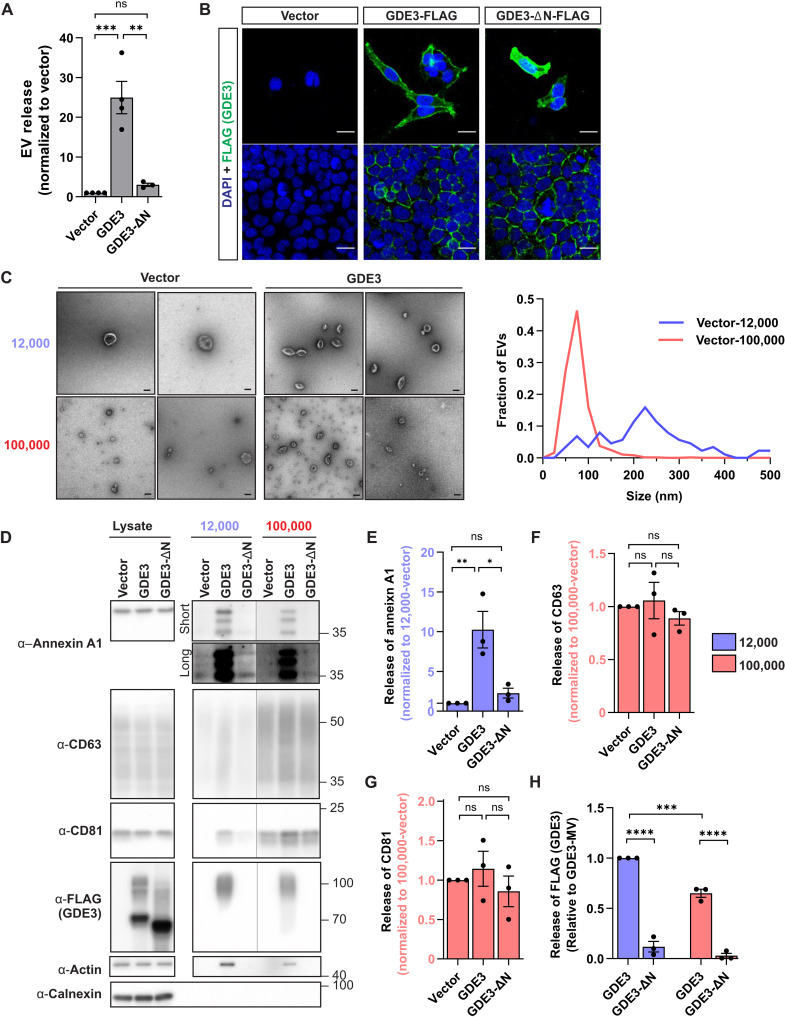
GDE3 induces the release of MVs in HEK293T cells. (**A**) Graph quantifying normalized EV concentration from transfected HEK293T cells. (**B**) Representative image of HEK293T cells stably expressing GDE3 or transfected GDE3-ΔN. FLAG (GDE3, green) and DAPI (blue). Scale bar, 20 μm. (**C**) Representative electron microscope (EM) images of 12,000 and 100,000 EVs from HEK293T cells transfected with vector or plasmids expressing GDE3. Scale bar: 100 nm. Histogram of sizes of 12,000 and 100,000 EVs from vector-transfected HEK293T cells visualized by EM. (**D**) Representative Western blots and graphs (**E** to **H**) quantifying 12,000 and 100,000 EVs from HEK293T cells transfected with the indicated plasmids. Long and short refer to exposure times. (E) MV marker annexin A1 was quantified in 12,000 EVs; exosome markers CD63 (F) and CD81 (G) were quantified in 100,000 EVs [see fig. S1 (C to E) for additional quantification]; (H) GDE3 (FLAG) was quantified in 12,000 and 100,000 EVs. All graphs are means ± SEM. Data points are biological replicates. ns, *P* > 0.05; *P < 0.05, **P < 0.01, ***P < 0.001, and ****P < 0.0001. See table S1 for statistical details. ns, not significant.

A previous study showed that GDE3 increased the release of the GPI-AP CNTFRα in EVs (*13*), but the mechanism for this observation is not known. To explore the role of GDE3 in EV release more deeply, we transfected GDE3 into human embryonic kidney (HEK) 293T cells and isolated EVs from the medium after 24 hours using a differential centrifugation strategy (*17*). Measurements of EV release by nanoparticle tracking analysis (NTA) show that GDE3 overexpression in HEK293T cells increased the numbers of EVs by 25-fold (Fig. 1A, fig. S1A, and table S1). GDE3-ΔN is a mutant form of GDE3 that lacks the 38–amino acid N-terminal domain and fails to release CNTFRα in EVs despite being localized to the cell surface at similar amounts as GDE3 and enzymatically active (Fig. 1B) (*13*). Overexpression of GDE3-ΔN did not increase the release of EVs (Fig. 1A and fig. S1A), indicating that the N-terminal domain is required for EV release. Moreover, the failure of GDE3-ΔN to release EVs suggests that GDE3 EV release activity is regulated by a designated domain and not simply a consequence of increased amounts of GDE3 on the plasma membrane. In addition, because GDE3-ΔN can still cleave GPI-APs (*13*), its inability to release EVs is unlikely to be a consequence of GPI-AP cleavage or GPI-AP surface levels. Together, these observations indicate that GDE3 robustly induces the release of EVs through mechanisms that are dependent on its N-terminal domain.

GDE3 is expressed on the plasma membrane (Fig. 1B) (*13*, *14*). Because this is the site of MV generation, we hypothesized that GDE3 primarily releases MVs and not exosomes. MVs are typically heterogeneous in size and are generally larger than exosomes (*18*); we exploited this size difference using differential centrifugation to generate a preparation of EVs that primarily contains MVs and one that contains MVs and exosomes (fig. S1B) (*18*). Larger EVs, typically MVs, are sedimented by centrifugation at 12,000*g* (12,000 EVs), while smaller EVs are sedimented by centrifugation at 100,000*g* (100,000 EVs) (fig. S1B). Electron microscopy shows that EVs isolated by this method have typical cup-shaped morphology (Fig. 1C) and distinct sizes. The 12,000 EVs have a broad size range peaking at 225 nm, matching the size distribution of MVs, while the 100,000 EVs have a narrow distribution peaking at 75 nm, matching the distribution of exosomes (Fig. 1C). Western blots demonstrate the absence of the endoplasmic reticulum marker calnexin, confirming that EV preparations are free from cellular membrane contamination (Fig. 1D) (*19*). The 12,000 EVs contained the MV marker annexin A1 (*18*, *20*) but were depleted of the exosomal markers CD63 and CD81 (Fig. 1D and fig. S1, C to E), while the 100,000 EVs contained both annexin A1 and CD63/CD81 (Fig. 1D and fig. S1, C to E). This indicates that 12,000 EVs contain primarily MVs, while 100,000 EVs contain smaller MVs and exosomes.

To distinguish the type of EV that GDE3 releases, we expressed GDE3 or GDE3-ΔN in HEK293T cells, isolated EVs by differential centrifugation, and performed Western blotting for MV and exosome markers. Expression of GDE3 in HEK293T cells increased the amount of the MV marker annexin A1 (*18*, *20*) by 10-fold in 12,000 EVs (Fig. 1, D and E) and, although not reaching statistical significance, elevated annexin A1 in 100,000 EVs (fig. S1C). However, GDE3 expression did not affect the amounts of the exosome markers CD63 or CD81 in 100,000 EVs (Fig. 1, D, F, and G) and in 12,000 EVs (fig. S1, D and E). CD81 levels in 12,000 EVs showed an increasing, but nonsignificant, trend with GDE3 expression, possibly reflecting the incorporation of CD81 on MVs from the population of CD81 reported to be expressed on the plasma membrane (fig. S1E) (*21*). Consistent with an inability to release exosomes, measurement of exosome numbers through a microchip antibody capture-based platform found that equivalent numbers of CD63^+^, CD81^+^, and CD9^+^ EVs were produced by GDE3 expression in HEK293T cells (fig. S1F). Together, these observations suggest that GDE3 predominantly releases MVs and not exosomes.

GDE3 itself is released in EVs. Western blots of isolated EVs from transfected HEK293T cells detect GDE3 expression in 12,000 EVs and, to a lower extent, in 100,000 EVs (Fig. 1, D and H), likely reflecting that GDE3 is released in large and small MVs. GDE3 exists in a 70-kDa form and a glycosylated 100-kDa form within HEK293T cells, but only the 100-kDa form of GDE3 is released in EVs, implying some glycosylation-dependent regulation of release (Fig. 1D). To verify that GDE3 is released in EVs, we used size exclusion chromatography (SEC), a method that separates EVs from EV-like particles and protein aggregates. GDE3 is present in particles of a similar size to particles containing CD63, an established marker for EVs, confirming that GDE3 is released on EVs and not as an aggregate nor on a non-EV particle (fig. S1G). Compared to CD63, GDE3 tends to be present in earlier fractions that correspond to larger EVs, consistent with the notion that GDE3 is released in MVs. Supporting the idea that GDE3 is released on MVs, we detected EVs that contained GDE3 and endogenous annexin A1 by immunohistochemical staining of 12,000 EVs purified from HEK293T cells transfected with plasmids expressing GDE3 (fig. S1H). Together, these observations suggest that GDE3 is capable of releasing a subtype of EVs from the plasma membrane that contains annexin A1 and GDE3.

### GDE3 is necessary for MV release in astrocytes

**Fig. 2.**
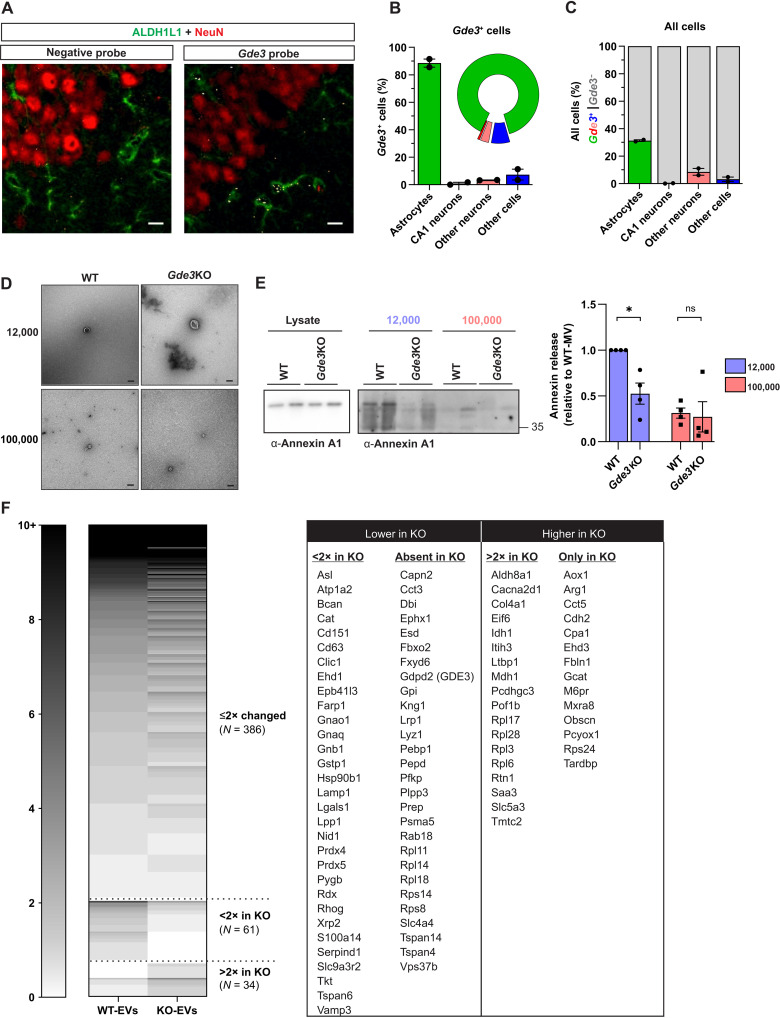
GDE3 is required for the release of annexin A1^+^ EVs in astrocytes. (**A**) Representative confocal images of P14 wild-type (WT) CA1 hippocampal sections showing *Gde3* mRNA (white) with astrocytic ALDH1L1 (green) and neuronal NeuN (red) markers. Scale bar, 10 μm. (**B**) Quantification of *Gde3*-expressing cells by cell type. (**C**) Fraction of each cell type that expresses GDE3. (**D**) Representative electron microscopy images of 12,000 and 100,000 EVs from WT and *Gde3* knockout (KO) astrocytes transfected with plasmids expressing GDE3. Scale bar, 100 nm. (**E**) Representative Western blot and quantification of annexin A1 released in 12,000 EVs and 100,000 EVs by WT and *Gde3* KO astrocytes. (**F**) Heatmap showing the number of detected peptides for each protein identified in EVs from WT and *Gde3* KO astrocytes. Proteins are grouped by their change between genotypes. See table S2 for a comprehensive list of differential proteins. All graphs are means ± SEM; data points refer to biological replicates. ns, *P* > 0.05; *P < 0.05. See table S1 for statistical details.

In the embryonic spinal cord, *Gde3* is expressed in OPCs and astrocytes (*13*), and cell-specific transcriptomic and expression studies (*22*–*25*) identify *Gde3* as expressed primarily in astrocytes in the developing and adult brain. We confirmed these observations by quantitative polymerase chain reaction (qPCR) of cells acutely isolated from P14–15 (postnatal days 14 and 15) mouse hippocampus. Using magnetic beads coated with antibodies against cell-specific surface antigens, we separated astrocytes and neurons from other cells with high affinity (*26*) as assayed by the expression of the astrocyte marker *Aldh1l1* and the neuronal marker *Rbfox3* (NeuN) (fig. S2A). *Gde3* (*Gdpd2*) RNA was enriched in astrocytes compared to neurons confirming that the primary site of *Gde3* expression is in astrocytes (fig. S2A). To determine on a cell-by-cell basis where *Gde3* is expressed, we performed RNA in situ hybridization using RNAscope in the P14 mouse hippocampus (Fig. 2, A to C). *Gde3-*expressing cells are primarily astrocytes (88%) (Fig. 2B), and of all astrocytes, *Gde3* expression corresponds to approximately 30% of astrocytes, indicating that it labels a subset of astrocytes in the hippocampus (Fig. 2C). Almost no CA1 neurons express *Gde3* (<1%), and very few other neurons (3%) expressed *Gde3* (Fig. 2C). RNAscope detected a few cells of the undefined type that expressed *Gde3* (Fig. 2, B and C), which likely correspond to OPCs. These analyses, together with published datasets, confirm that *Gde3* is primarily expressed in astrocytes.

To determine whether GDE3 is required for MV release in astrocytes, we prepared purified cultures of astrocytes from P5 *Gde3* knockout (KO) and wild-type (WT) pups; these cultures were reproducibly enriched for ALDH1L1^+^ astrocytes (>95%) with the minimal presence of other cell types (fig. S2B). We isolated EVs using the same methodologies used for HEK293T cells (fig. S1B) and confirmed that astrocytic EVs showed similar cup-shaped morphology as HEK293T preparations (Fig. 2D). Western blot of isolated EVs found a 48% reduction in the amount of annexin A1 in 12,000 EVs from *Gde3* KO astrocytes compared with WT and no difference in 100,000 EVs, showing that GDE3 is required for the release of MV associated cargo from astrocytes (Fig. 2E). We observed GDE3 expression in 30% of astrocytes (Fig. 2C), yet loss of GDE3 affects nearly half of annexin A1 release in MVs (Fig. 2E). This suggests that GDE3^+^ astrocytes release a disproportionate amount of annexin A1, consistent with GDE3 being a major driver of MVs containing annexin A1 from astrocytes.

To gain deeper insight into the protein cargoes of MVs released by GDE3, we isolated EVs from WT- and *Gde3* KO–cultured astrocytes by SEC and compared their protein composition by mass spectrometry. EVs were purified by SEC to preserve the integrity of EVs and to remove non-EV components from the media, including small molecules, protein aggregates, soluble proteins, and many lipoprotein particles. A total of 481 proteins were identified in WT and *Gde3* KO EVs (table S2), and, of these, 51% of proteins corresponded to known EV proteins compiled in the mouse ExoCarta database (*27*) and 71 proteins were annotated as the top 100 abundant proteins in EVs in the Vesiclepedia database (*28*). Comparisons between EVs from WT and *Gde3* KO astrocytes identified 61 proteins that were twofold lower or completely absent in *Gde3* KO EVs and 34 proteins that were twofold higher or only present in *Gde3* KO EVs (Fig. 2F and table S2). Of note, GDE3 was detected in WT-EVs, but not in *Gde3* KO-EVs, confirming that endogenous GDE3 is released in EVs. Furthermore, annexin A1 amounts were reduced in *Gde3* KO EVs, corroborating our finding that annexin A1^+^ EVs are reduced when GDE3 function is ablated (table S2). These observations suggest that the composition of EV cargos is disrupted in the absence of GDE3 and provides supporting evidence that the GDE3 pathway is required in astrocytes for the release of multiple proteins in EVs, including annexin A1 and GDE3 itself.

It is notable that NTA studies showed that the total number of EVs was comparable between WT and *Gde3* KO conditions (fig. S2C). This indicates that either MVs generated by GDE3 are a small subset of total EV numbers or surrogate mechanisms maintain numerical EV homeostasis when GDE3-dependent release mechanisms are perturbed, a phenomenon observed with the manipulation of other EV biogenesis pathways (*29*). Regardless, while alternate pathways in astrocytes can maintain overall EV numbers, they are unable to faithfully compensate for the cargo and function of MVs released by GDE3. Viewed with our gain-of-function experiments in HEK293T cells, these observations suggest that GDE3 is endogenously required for the release of a specific subtype of MVs with distinct cargo that includes GDE3 and annexin A1.

### GDE3 promotes MV release by regulating WAVE3-dependent actin remodeling

**Fig. 3.**
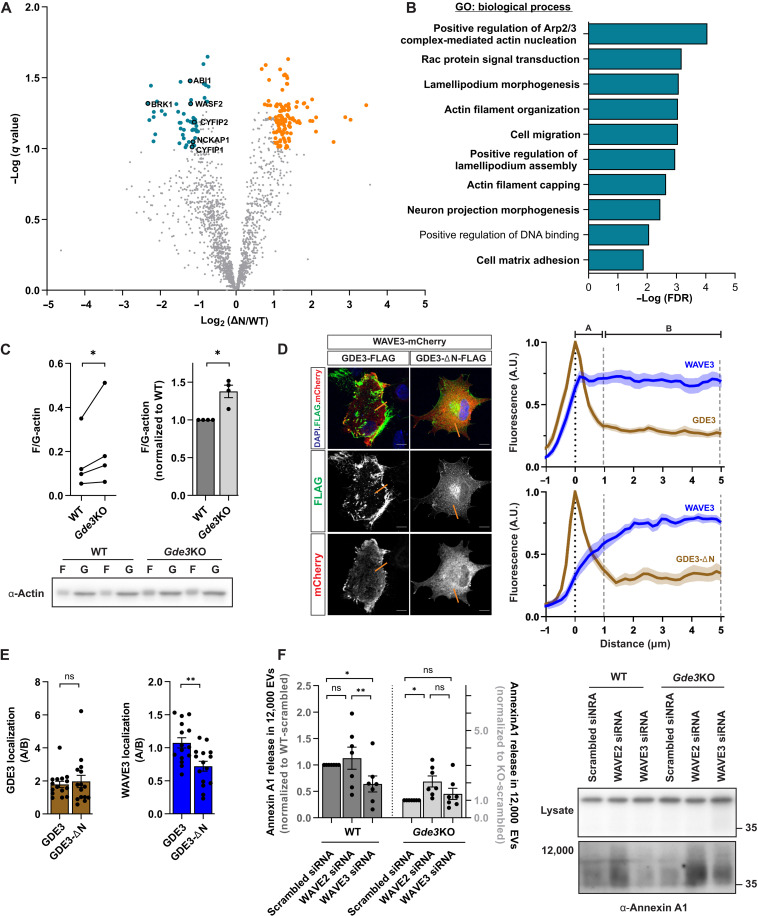
GDE3 remodels the actin cytoskeleton via WAVE3 to release MVs. (**A**) Volcano plot showing proteins with increased (teal dots) or decreased (orange dots) proximity to GDE3 compared to GDE3-ΔN. WRC components are highlighted. (**B**) False discovery rate (FDR) of the top 10 Gene Ontology (GO) terms in the Biological Process category from proteins with increased proximity to GDE3 [teal dots from (A)]. (**C**) Graphs quantifying F/G-actin polymerization ratio of WT and *Gde3* KO astrocytes. Left: F/G ratio. Right: Normalized ratios. Bottom: Representative Western blots of F-actin and G-actin. (**D**) Representative images of WT astrocytes expressing WAVE3-mCherry and GDE3 or GDE3-ΔN. Scale bars, 10 μm. Fluorescence intensity was quantified along orange lines and graphed. Plots show the average intensity of WAVE3, GDE3, or GDE3-ΔN along the distance of the line. A, surface region; B, intracellular region. (**E**) Bar graphs show the intensity of GDE3 (brown) and WAVE3 (blue) intensities between the surface (0 to 1 μm) and intracellular (1 to 5 μm) compartments of each cell. (**F**) Representative Western blots and graphs quantifying annexin A1 amount in 12,000 EVs from WT or *Gde3* KO astrocytes with the indicated siRNA treatments. All graphs are means ± SEM. Data points correspond to biological replicates in (C) and (F) and refer to individual cells in (E). ns, *P* > 0.05; *P < 0.05 and **P < 0.01. See table S1 for statistical details. A.U., arbitrary units.

The mechanism by which GDE3 releases MVs is not known. Our studies so far indicate that GDE3-ΔN fails to release EVs (Fig. 1, A and D to H, and fig. S1A and C to E), suggesting that the N terminus may serve as an interaction hub for other protein(s) that work with GDE3 to release MVs. To identify candidate mediators of GDE3 function, we used the TurboID proximity labeling system, which rapidly biotinylates proteins within a 10-nm radius (*30*). We expressed GDE3-TurboID and GDE3-ΔN-TurboID fusion proteins in HEK293T cells, purified biotinylated proteins, and analyzed them by mass spectrometry (fig. S3A). We identified 179 proteins that showed the differential proximal location to GDE3 and GDE3-ΔN (Fig. 3A and table S3), of which 62 were deemed closer to GDE3 compared to GDE3-ΔN (teal dots; Fig. 3A and table S3). Gene Ontology (GO) analysis revealed that these proteins are enriched for inclusion in “Extracellular Exosomes” [GO:0070062, false discovery rate (FDR) = 1.20 × 10^−7^; fig. S3B] and for functions related to the actin network and cytoskeleton regulation (Fig. 3B and fig. S3B).

Actin regulates MV release (*1*, *31*–*33*), and GDE3 is associated with changes in cell morphology and actin dynamics (*34*, *35*). We thus hypothesized that GDE3 promotes MV release by integrating with mechanisms that regulate the actin cytoskeleton. To test this hypothesis, we used a differential centrifugation assay that separates globular (G-actin) from polymerized actin (F-actin). A comparison of cultured astrocytes prepared from P5 pups showed that *Gde3* KO astrocytes had a higher ratio of F/G-actin compared with WT astrocytes, consistent with a role for GDE3 in regulating actin dynamics (Fig. 3C). The WRC (or SCAR complex) remodels the actin cytoskeleton by nucleating new Arp2/3 molecules onto existing actin chains to form new actin branches (*36*). Notably, our TurboID screen for proteins proximal to GDE3 identified all five components of the WRC (fig. S3B, teal box), of which WAVE proteins (WAVE1-3) are the central component (*36*, *37*). Immunohistochemical detection of GDE3, GDE3-ΔN, and WAVE2, which is ubiquitously expressed and was identified in our screen, showed colocalization at the plasma membrane in transfected HEK293T cells, validating the results of our screen (fig. S3C). In addition, WAVE3, which is enriched in astrocytes (*22*), colocalizes with GDE3 at the plasma membrane in cultured astrocytes (Fig. 3, D and E). However, WAVE3 localization at the plasma membrane was enhanced in GDE3-expressing cells compared to GDE3-ΔN (Fig. 3, D and E), suggesting that the N terminus may recruit WAVE2/3 to the plasma membrane in astrocytes. Notably, coimmunoprecipitation experiments in transfected HEK293T cells or cultured astrocytes did not detect complexes containing GDE3 and WAVE2 or WAVE3 (fig. S3, D and E), suggesting that GDE3-WAVE associations are transient or that they function within the same microdomain via an intermediary protein.

To determine whether GDE3 regulates the WRC to release MVs, we reduced WRC activity by knocking down WAVE2 and WAVE3 expression in astrocytes. Treatment with WAVE-specific small interfering RNAs (siRNAs) reduced the amount of WAVE2 and WAVE3 protein by approximately 50% in WT- and *Gde3* KO–cultured astrocytes compared with scrambled control siRNAs (fig. S4). WT and *Gde3* KO astrocytes expressed equal levels of WAVE2 and WAVE3 (fig. S4). Western blot analysis of 12,000 EVs showed that knockdown of WAVE3 in WT astrocytes reduced annexin A1 release by 36% (Fig. 3F), suggesting that WAVE3 is required for the release of annexin A1^+^ MVs from astrocytes. In contrast, WAVE2 knockdown did not perturb the amount of released annexin A1 (Fig. 3F). Of note, WAVE2 depletion resulted in an increased WAVE3 expression, which might compensate for decreases in WAVE2 activity (fig. S4). While we cannot rule out possible contributions of WAVE2 to MV release, these observations identify a nonredundant function for WAVE3 in the release of annexin A1^+^ MVs from astrocytes. In *Gde3* KO astrocytes, the knockdown of WAVE3 did not decrease annexin A1 release further than the reduction associated with the loss of GDE3 (Fig. 3F), suggesting that WAVE3 and GDE3 operate within the same pathway to promote the release of annexin A1^+^ MVs in astrocytes. These studies identify GDE3 regulation of WAVE3 as a physiological regulator of annexin A1^+^ GDE3^+^ MVs from astrocytes through modulation of actin dynamics. In *Gde3* KO astrocytes, the knockdown of WAVE2 led to an increase in annexin A1 release (Fig. 3F), suggesting possible inhibitory roles for WAVE2 or compensatory activity by WAVE3 via other mechanisms when GDE3 is ablated.

### GDE3 astrocytic expression regulates postsynaptic neuronal function

We next sought to determine whether the EVs released by the GDE3-WAVE3 pathway in astrocytes have physiological functions. Astrocyte numbers in vivo were not altered between WT and *Gde3* KO (fig. S5A), and Western blot analysis of protein extracts prepared from WT and *Gde3* KO P12–15 hippocampus and cortex found no change in the expression of the glutamate transporters excitatory amino acid transporter 1 (EAAT1) (GLAST) and EAAT2 (GLT1), the lactose transporter monocarboxylate transporter 4 (MCT4), and GFAP (glial fibrillary acidic protein) (fig. S5B) (*38*, *39*). Furthermore, transmission electron microscopy of P12–15 hippocampus showed no major defects in morphology and coverage of astrocytes around synapses in WT and *Gde3* KO animals (fig. S5C). While not a comprehensive examination of astrocyte function, these results suggest that GDE3 ablation does not cause widespread disruptions in astrocyte number, lactose and glutamate transporters, and gross morphology.

**Fig. 4.**
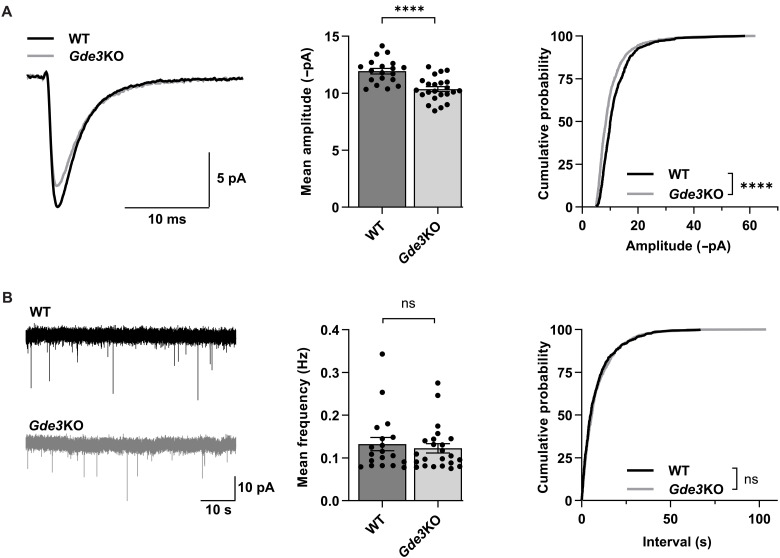
GDE3 regulates neuronal postsynaptic function. (**A** and **B**) Measurements in P12–15 WT and *Gde3* KO CA1 cells. (A) Amplitudes of mEPSCs. Left: Superimposed representative averaged traces aligned by rise time. Middle: Graph quantifying the mean amplitude of each cell. Right: Cumulative distribution of amplitudes. (B) Frequency of mEPSCs. Left: Representative raw recording traces over 1 min. Middle: Graph quantifying the mean frequency of each cell. Right: Cumulative distribution of inter-event interval of events. All bar graphs are means ± SEM; data points refer to cells from six WT and eight *Gde3* KO mice, and males and females were used in both groups. *P* > 0.05 (ns); ****P < 0.0001. See table S1 for statistical details.

Astrocytic EVs are known to affect diverse aspects of neuronal structure and function (*11*), raising the possibility that EVs released by the GDE3-WAVE3 pathway in astrocytes are required for appropriate neuronal function. In support of this notion, our proteomic analysis of EV cargoes identified astrocytic proteins enriched in the “Synapse” (GO:0045202), specifically the “Postsynapse” (GO:0098794) (table S2), suggesting potential roles for GDE3 EVs in astrocyte-neuronal communication in the context of synaptic biology. To test this hypothesis, we performed whole-cell patch-clamp recording experiments on CA1 hippocampal neurons prepared from slices of male and female P12–15 WT and *Gde3* KO mice. We detected a significant reduction in the magnitude of miniature excitatory postsynaptic current (mEPSC) amplitudes in *Gde3* KO animals compared with WT (Fig. 4A), but no change in the frequency (Fig. 4B). Changes in amplitude are typically a result of postsynaptic changes in glutamate receptor number or properties, while the frequency of mEPSC events reflects presynaptic changes that affect the rate of synaptic vesicle fusion. These observations suggest that GDE3 is required for appropriate postsynaptic responses but not presynaptic function. Kinetics of the mEPSC waveform (fig. S6A) and series and membrane resistances (fig. S6B) were equivalent between WT and *Gde3* KO animals, indicating that the difference in mEPSC amplitude was not due to intrinsic property changes of neurons. Amplitudes of each genotype remained constant across the P12–15 experimental window (fig. S6C). In addition, no ultrastructural changes in synapses were observed between genotypes (fig. S5C). GDE3 is not expressed in CA1 neurons (Fig. 2, A to C, and fig. S2A), and hippocampal mEPSC recordings from P12–15 mice that are conditionally deleted for *Gde3* in neurons show no change in mEPSC amplitudes or frequencies compared with controls (fig. S7). These observations indicate a non–cell-autonomous function for GDE3 and raise the possibility that astrocytic EVs released by GDE3 are required to regulate the amplitude of neuronal postsynaptic glutamatergic responses.

### GDE3-WAVE MVs rescue synaptic response in *Gde3* KO animals

**Fig. 5.**
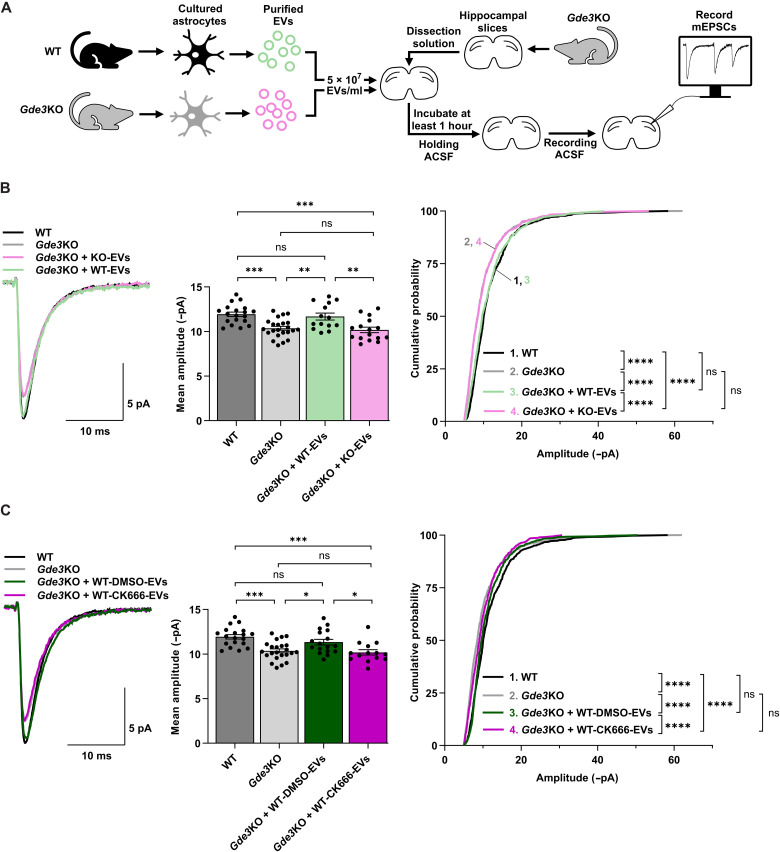
GDE3-WAVE EVs restore postsynaptic function in *Gde3* KO slices. (**A**) Schematic of protocol for (B). (**B** and **C**) Measurements of mEPSC amplitudes in P12–15 *Gde3* KO CA1 cells treated with EVs. WT and *Gde3* KO data in (B) and (C) (gray bars, gray and black curves) are reproduced from Fig. 4A. Left: Superimposed representative averaged traces aligned by rise time. Middle: Graph quantifying the mean amplitude of each cell. Right: Cumulative distribution of amplitudes. (B) EVs were added from WT (WT-EVs, green) and *Gde3* KO (KO-EVs, pink) astrocytes. (C) EVs were added from WT astrocytes treated with dimethyl sulfoxide (DMSO) (WT-DMSO-EVs, dark green) and 100 μM CK666 (WT-CK666-EVs, dark pink). All bar graphs are means ± SEM; data points correspond to cells from four mice treated with WT-EVs, three mice treated with KO-EVs, 4 mice treated with DMSO-EVs, and 3 mice treated with CK666-EVs; at least three independent EV isolations were used for each group. ns, *P* > 0.05; *P < 0.05, **P < 0.01, ***P < 0.001, and ****P < 0.0001. See table S1 for statistical details.

To determine whether EVs released by GDE3 mediate the non–cell-autonomous effects of GDE3 on postsynaptic neuronal responses, we repeated our mEPSC recordings, this time with the addition of astrocytic EVs to hippocampal slices. We isolated EVs from the medium of WT astrocytes (WT-EVs) or *Gde3* KO astrocytes (KO-EVs) using SEC. We used SEC instead of ultracentrifugation for our slice experiments for the reasons described earlier. SEC will isolate both exosomes and MVs (*40*); however, *Gde3* KO EVs will lack the MV subtype released by GDE3 (Fig. 2, E and F). We incubated slices from *Gde3* KO animals with 5 × 10^7^ EVs/ml of WT-EVs or KO-EVs for at least 1 hour, transferred slices to fresh recording solution, and performed whole-cell recordings (Fig. 5A). The addition of WT-EVs to *Gde3* KO slices restored amplitudes of neuronal mEPSCs to WT levels, while the addition of KO-EVs had no effect (Fig. 5B). Incubation with WT-EVs longer than 1 hour resulted only in mild increases in effect size (fig. S8A) suggesting that the majority of the effect happens within the first hour, and elevated amplitudes were maintained for at least 1.5 hours after incubation with EVs (fig. S8B). The addition of WT-EVs to WT slices did not affect the amplitude or frequency of mEPSCs (fig. S8C), suggesting that GDE3-mediated effects in WT slices are saturated. In addition to the genetic ablation of GDE3-EVs, we used a pharmacological approach to blocking the production of GDE3 MVs. We treated WT astrocytes with CK666, a specific pharmacological inhibitor of Arp2/3 activity (*41*), the downstream target of the WRC. While CK666 will block the activity of all WAVEs and other enzymes that remodel Arp2/3, WAVE3 appears to be the major mediator of MVs released by GDE3 (Fig. 3F). EVs from WT astrocytes treated with dimethyl sulfoxide (DMSO) (WT-DMSO-EVs) restore mEPSC amplitudes in *Gde3* KO slices (Fig. 5C). In contrast, EVs from WT astrocytes treated with 100 μM CK666 (WT-CK666-EVs) failed to restore mEPSC amplitudes in *Gde3* KO slices (Fig. 5C), similar to KO-EVs. Notably, EVs from all four EV conditions showed no effects on the frequency of mEPSCs (fig. S8, D and E), highlighting the compartment and functional specificity of EVs released by GDE3. Furthermore, EVs had no effects on intrinsic neuronal properties, evident by comparable waveform kinetics (fig. S8F) and series and membrane resistances between conditions (fig. S8G). These observations support the model that the GDE3-WAVE3 pathway regulates the production of MV subtypes from astrocytes with shared functional properties that are transmitted to neurons to regulate postsynaptic neuronal function.

### GDE3 acts through mGluR1/5 to regulate neuronal function

**Fig. 6.**
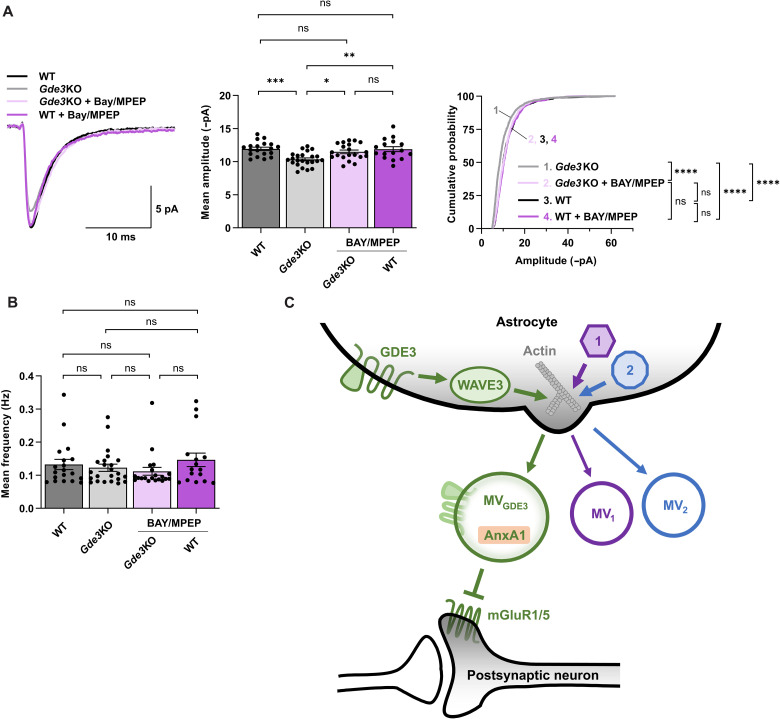
GDE3 regulates postsynaptic responses via mGluR1/5. (**A** and **B**) Measurements of mEPSC amplitudes in P12–15 *Gde3* KO CA1 cells treated with Bay/MPEP. (A) Left: Superimposed representative averaged traces aligned by rise time. Middle: Graph quantifying the mean amplitude of each cell. Right: Cumulative distribution of amplitudes. WT and *Gde3*KO data are reproduced from Fig. 4A. (B) Graph quantifying the mean frequency of each cell. WT and *Gde3*KO data are reproduced from Fig. 4B. (**C**) Schematic of a model that posits that GDE3-WAVE3 is one of several pathways in astrocytes that integrate with the actin network to generate distinct MV subtypes with unique functions. GDE3 regulates WAVE3 to remodel the actin cytoskeleton to release MVs from the plasma membrane that contains GDE3 and annexin A1 (green, MV_GDE3_). MV_GDE3_s act on postsynaptic neurons to regulate postsynaptic responses through the inhibition of mGluR1/5. We hypothesize that alternative MV release pathways shown in purple and blue (1 and 2) can remodel actin to independently release distinct MV subtypes (MV_1_ and MV_2_) that have separate functions unrelated to MV_GDE3_. Thus, designated EV release pathways can independently regulate general mechanisms of EV release to control EV functional outputs. All bar graphs are means ± SEM; data points correspond to cells from six WT animals and four *Gde3* KO animals treated with Bay/MPEP. ns, *P* > 0.05; *P < 0.05, **P < 0.01, ***P < 0.001, and ****P < 0.0001. See table S1 for statistical details.

We next sought to understand how GDE3-EVs act on neurons to regulate postsynaptic function. We hypothesized that GDE3-EVs act on the postsynaptic group I metabotropic glutamate receptors mGluR1 and mGluR5. When activated by glutamate, these receptors can cause the endocytosis of AMPA receptors, which leads to a reduction in postsynaptic strength (*42*). We hypothesize that, in the absence of GDE3, the mGluR1/5 pathway is abnormally activated in neurons, resulting in decreased postsynaptic amplitude. Bay (Bay-36-7602) (*43*) and MPEP (2-methyl-6-(phenylethynyl)pyridine) (*44*) are established and validated inhibitors of mGluR1/5 signaling that block the basal activity of these receptors (*45*). To test this hypothesis, we treated WT and *Gde3* KO hippocampal slices from P12–15 mice with Bay/MPEP and performed whole-cell patch-clamp recordings from CA1 neurons. We found that Bay/MPEP restored the amplitude of mEPSCs in *Gde3* KO slices to WT levels (Fig. 6A). Bay/MPEP had no effect on mEPSC amplitudes in WT slices (Fig. 6A) and did not alter mEPSC event frequency in WT or *Gde3* KO slices (Fig. 6B). These observations suggest that the mGluR1/5 pathway is overactive in the CA1 neurons of *Gde3* KO mice and imply that GDE3-EVs may restore amplitude by inhibiting mGluR1/5 signaling.

## DISCUSSION

EVs are emerging as important mediators of intercellular communication, but how cells control the complex signaling output of EVs remains unclear. Here, we provide evidence that distinct molecular pathways feed into global pathways of EV biogenesis to selectively promote the release of specific EV subtypes with specialized functional properties. We find that GDE3 is necessary and sufficient to release a subset of molecularly defined MVs from the plasma membrane through WAVE3, a component of the WRC that regulates the actin cytoskeleton, a known mediator of MV release. GDE3 is primarily expressed in astrocytes, and astrocytic MVs released by the GDE3-WAVE3 pathway are required for appropriate postsynaptic responses, likely through the modulation of the mGluR1/5 pathway. Thus, GDE3 constitutes a regulatory mechanism that modulates the actin cytoskeleton via WAVE3 to induce the formation of a distinct subset of MVs that have compartment-specific functions in the postsynapse (Fig. 6C). These observations suggest that the generation of functionally distinct sets of EVs can be implemented via specific molecular pathways that fine-tune downstream cellular events important for EV biogenesis.

We show here that EVs generated by GDE3-WAVE3 are molecularly and functionally distinct from other EVs generated by astrocytes. GDE3-WAVE3 EVs are MVs that bud from the plasma membrane and are defined by the presence of annexin A1 and GDE3. In terms of function, MVs released by GDE3 are required for appropriate postsynaptic responses and do not affect presynaptic release frequency, suggesting specific and compartmentalized roles for GDE3-WAVE3 MVs. The addition of non–GDE3-WAVE3 EVs, generated by genetic or pharmacological manipulation of astrocytes, was unable to rescue synaptic deficits in *Gde3* KO animals, highlighting the functionality of GDE3-WAVE3 MVs. These collective observations suggest that the EVs produced by the GDE3-WAVE3 release pathway are specialized and nonredundant for appropriate postsynaptic responses. Our studies involve the use of astrocytes cultured in the presence of serum that can influence astrocyte biology (*46*); nonetheless, the ability of WT but not Gde3 KO nor CK666-WT astrocytic EVs to specifically rescue the postsynaptic deficits in *Gde3* KO slices underscores the involvement of the GDE3-WAVE3 pathway in this system. Because GDE3-EVs are molecularly defined, are released by a particular biogenesis pathway, and have a specific biological function, we propose that GDE3-EVs can be viewed as a unique EV subtype.

Our finding that GDE3 mediates MV release via WAVE3 in astrocytes helps clarify the general question of how different types of EVs are generated and regulated within cells. Modulation of the actin cytoskeleton is known to be involved in many forms of MV release. For example, MV fission and release from the plasma membrane require actin and myosin interactions that are mediated in part by ARF6 and ARF1 (*1*, *9*). These interactions result in the activation of the contractile machinery at the plasma membrane required for MV generation (*47*). WAVE3 is one of three isoforms of the WRC, a major regulator of the actin cytoskeleton (*37*). Our studies implicate actin-based processes via WAVE3 as important regulators of MV release from astrocytes. WAVE3 appears to be a major driver of annexin A1^+^ MVs in astrocytes because knocking down of WAVE3 by approximately 50% leads to a 45% reduction of annexin A1 release in MVs, indicating that WAVE3 release function is nonredundant. Astrocytes also express WAVE2. The requirement for WAVE2 in annexin A1 release is less clear but, at minimum, appears minor in comparison to WAVE3, suggesting that annexin A1^+^ MVs release function is primarily dependent on WAVE3 and not broadly associated with generalized actin regulation. This finding suggests that select pathways are likely to converge on the actin network to regulate the release of different EV subtypes, perhaps through the selective use of other proteins that regulate actin dynamics, such as other WAVE isoforms, ARFs, or myosins. On the basis of our observations, we suggest a model where dedicated molecular pathways intersect with general mechanisms of EV release to confer the selectivity of EV production (Fig. 6C). While this model focuses on actin, it may also apply to other EV biogenesis pathways, such as the ceramide pathway or ESCRT. Another concept that emerges from our work is that the restricted expression of components of the release pathway is a major contributor to the specificity of EV subtype production. GDE3 and WAVE3 are enriched in astrocytes, and surveys of gene expression databases across neural and non-neural cell types reveal an unexpected restriction of GDE3 and WAVE3 coexpression to astrocytes and Muller glia. This expression pattern provides a regulatory module for GDE3-WAVE3 MV release that is localized to astrocytes and potentially Muller glia. Therefore, in addition to the specificity imposed by the selective regulation of actin remodeling, the biogenesis of different EV subtypes can be coordinated by the restricted expression of critical components of the release pathway across different axes such as cell type and potentially time and space.

Our proteomic analysis (Fig. 2F) reveals notable but limited differences between the protein content of WT EVs and *Gde3* KO EVs, suggesting that specific cargos are recruited by the GDE3-WAVE3 pathway into EVs. One, or several of these cargoes, is hypothesized to inhibit mGluR1/5 signaling to regulate postsynaptic responses. This raises two important questions. First, what is the mechanism by which unique cargoes are recruited into EVs by GDE3-WAVE3? How this occurs is unclear; however, we speculate that GDE3 may act as a hub for pathways important for cargo recruitment via direct/indirect physical associations or through co-compartmentalization at sites of EV biogenesis, for example, in select membrane microdomains. Second, what are the cargos in EVs released by GDE3-WAVE3 that mediate their function in postsynaptic responses? Prime candidates for future analysis include the proteins, miRNAs, mRNAs, or lipids that are differential in WT and *Gde3* KO EVs, in particular, those known to be involved in synapse biology (Fig. 2F). Of note, proteins released by astrocytes known to be important for synapse formation and maturation such as thrombospondins, glypicans 4 and 6, Hevin, and secreted protein acidic and rich in cysteine (*48*) were either not detected in our proteomic analysis of EVs or were not differential in their distribution between WT and *Gde3* KO EVs (table S2). WAVE proteins are also not detected in EVs, suggesting that GDE3-WAVE3 activity is required for MV release but not for GDE3 EV-mediated regulation of postsynaptic function. We note that annexin A1 and GDE3 are components of GDE3 MVs. Annexin A1 is unlikely to regulate postsynaptic function because approximately 50% of annexin A1 release is mediated by pathways other than GDE3, and we predict that the cargo required for the appropriate postsynaptic function is exclusive to or strongly enriched in GDE3 EVs. GDE3, however, is a potential candidate due to its unique biology. GDE3 can metabolize LPI to 2-arachidonoylglycerol (*15*, *16*), a ligand for cannabinoid receptors (CB1/2) on neuronal membranes (*49*). Therefore, GDE3 could regulate neuronal function by modifying the lipid composition of EVs or neurons that have incorporated GDE3 via EVs. GDE3 can also cleave GPI-APs off the surface of EVs (*13*), suggesting that GDE3-EVs could regulate neuronal function indirectly by providing a platform for GPI-APs to be released in proximity to a neuron. A better understanding of the mechanisms of cargo recruitment and function will be important to shed light on the functional complexity and plasticity of EVs.

Together, our studies have identified a specific EV biogenesis pathway that integrates with known release machinery to generate a subtype of EVs with distinct functions. The benefits of this type of regulatory mechanism are illustrated by our observation that the effects of GDE3 EVs on mEPSC amplitudes occur within an hour of application, a timeframe that is considerably shorter than the 24 hours or more examined in many studies of EV-mediated communication between astrocytes and neurons (*2*, *50*–*52*). Thus, EV subtypes released by GDE3-WAVE3 may operate on different time scales from other EVs. It is conceivable that GDE3-WAVE3 could be regulated to fine-tune the release of these specialized EVs according to temporal or context-dependent signals, while global EV homeostasis is maintained. Accordingly, we propose that the regulated release of functionally distinct EV subtypes through designated pathways would provide an effective means to coordinate a complex medley of EV-mediated responses to meet the dynamic physiological needs of cells.

## METHODS

### Animals

Animals were maintained and used in accordance with approved Johns Hopkins University IACUC protocols. *Gde3* KO mice were generated as described (*13*). Because *Gdpd2* (*Gde3*) is located on the X chromosome, WT/KO littermates can only be obtained in males. Therefore, for consistency, males (*Gde3^+/y^* or *Gde3^−/y^*) were used for all experiments in this study unless noted in the text or figure legends. *Nex-Cre* animals were generated and validated as described (*53*). Genotype was confirmed for all experimental animals.

### Animal perfusions

Animals were anesthetized with Avertin [PBS (phosphate-buffered saline), 1% 2,2,2 tribromoethanol, and 0.6% tert-amyl alcohol] and perfused with 20 ml of ice-cold 0.1 M phosphate buffer (pH 7.4) followed by 20 ml of ice-cold 4% paraformaldehyde (PFA) (MP Biochemicals, 150146) in phosphate buffer at a flow rate of ~10 ml/min. Brains were removed and incubated in 4% PFA overnight. Fixed tissue was embedded in paraffin and sectioned by the Johns Hopkins Reference Histology Core.

### Antibodies

All antibodies used in this study are listed here, and concentrations used for specific experiments are listed in the relevant subsections as follows: α-actin (Millipore, MAB1501R), α-actin (Cytoskeleton Inc., AAN02), α-ALDH1L1 (Abcam, ab177463), α–annexin A1 (Abcam, ab214486), α-calnexin (BD Biosciences, 610523), α-CD63 (Santa Cruz Biotechnology, 5275), α-CD81 (BD Biosciences, 555675), α-FLAG (Millipore, F7425), α-FLAG-M2 (Millipore, F1804), α-GAPDH (α–glyceraldehyde-3-phosphate dehydrogenase; Cell Signaling Technology, 8884), α-GFAP (Millipore, MAB360), α-GFAP (DAKO, Z0334), α-GLAST (*39*), α-GLT1 (*39*), α-MCT4 (Proteintech, 22787-1-AP), α-NeuN (Millipore, MAB377), α-RFP (α–red fluorescent protein; Abcam, ab62341), α-S100β (DAKO, Z0311), α-WAVE2 (Cell Signaling Technology, 3659), and α-WAVE3 (Cell Signaling Technology, 2806).

### Tissue dissection

P14-P15 animals were anesthetized with Avertin. Hippocampi and cortices were separated and placed in radioimmunoprecipitation assay (RIPA) buffer [150 mM NaCl, 50 mM tris, 1% NP-40, 0.5% DOC (deoxycholate), and 0.1% SDS (pH 7.5)] with protease inhibitors (Sigma-Aldrich, P8340). Tissue was lysed using sonication (30% power with 10 × 0.5–s pulses) and spun at 20,000*g* for 30 min to remove insoluble material. Protein was quantified using a BCA Protein Assay Kit (Thermo Fisher Scientific, 23225), and 12 μg was mixed with GLB [gel loading buffer, 50 mM tris, 2% SDS, 10% glycerol, bromophenol blue, and 0.1% β-mercaptoethanol (BME) (pH 6.8)] and frozen for subsequent Western blotting.

### Acute isolation of cells using Miltenyi beads

Isolation of cell types was based on a protocol from (*26*). Briefly, hippocampi of three to four P14–15 WT mice were dissected and pooled. The Neural Tissue Dissociation Kit with Papain (Miltenyi Biotec, 130-092-628) was used to generate single-cell suspensions following the manufacturer’s protocol for manual dissociation. Cells were separated by type using a series of bead-based sorting steps following the manufacturer’s protocol. Briefly, myelin was removed by incubating cells with Myelin Removal Beads II (Miltenyi Biotec, 130-092-731), and then applied to an MS Column (Miltenyi Biotec, 130-042-201). Flow-through from this step was used for subsequent steps. Astrocytes were isolated by incubating cells with Anti-ACSA-2 MicroBeads (Miltenyi Biotec, 130-097-678), and then applied to an MS Column. Flow-through from this column was collected for later use. Astrocytes were eluted off the column, and then allowed to flow through another MS Column for enhanced purity. Neurons were isolated from the flow-through that was collected earlier. These cells were incubated with non-neuronal antibody cocktail (Miltenyi Biotec, 130-126-602), and then applied to an MS Column. Flow-through containing the neurons was collected, and the remaining other cells were eluted off the column. Both these cell populations were separately applied to another MS Column to enhance their purity. Astrocyte and neuronal populations were pelleted and lysed in 250 μl of TRIzol (Thermo Fisher Scientific, 15596026) and stored at −80°C for later use.

For neuronal conditional *Gde3* KO experiments using *NexCre* animals, embryonic day (E)13.5 cortices and hippocampi from two pups were pooled. Single-cell suspensions were g enerated as above, but only the non-neuronal antibody cocktail beads were used with one application to an MS column. PCR conditions are described in (*13*). The following primers were used: Forward: 5′ GGAACATGACATTGCCTCCT; and Reverse: 5′ TAAAACCGAAGTCCCCCTTT.

### qPCR

RNA was extracted from TRIzol following the manufacturer’s protocol. A total of 300 to 400 ng of RNA was used for cDNA synthesis using High-Capacity cDNA Reverse Transcription Kit (Thermo Fisher Scientific, 4368814) following the manufacturer’s protocol. qPCR reactions were performed with Fast SYBR Green Master Mix (Thermo Fisher Scientific, 4385612) following the manufacturer’s protocol and measured with an Applied Biosystems StepOne Plus machine in technical triplicates. mRNA levels were normalized to *Gapdh*.

Primers used for qPCR are listed here. *Gdpd2*-F: 5′ CCAGCAAGTGCGACTGTATCT. *Gdpd2*-R: 5′ GACCAGGAGAGAGACGACCA. *Aldh1l1*-F: 5′ CAGGAGGTTTACTGCCAGCTA. *Aldh1l1*-R: 5′ CACGTTGAGTTCTGCACCCA. *Rbfox3*-F: 5′ GTAGAGGGACGGAAAATTGAGG. *Rbfox3*-R: 5′ GTGGGGTAGGGGAAACTGG. *Gapdh*-F: 5′ CGTCCCGTAGACAAAATGGT. *Gapdh*-R: 5′ TTGATGGCAACAATCTCCAC.

### RNAscope

Sections of P15 WT mouse brain were used for RNAscope using the Multiplex Fluorescent v2 Assay kit (ACDbio, 323110) and the RNA Protein Co Detection kit (ACDbio, 323180) according to the manufacturer’s protocol. Primary antibodies used for cell type identification: astrocytes with ALDH1L1 staining (1:40) and neurons with NeuN staining (1:500); appropriate secondary antibodies were used at 1:500. *Gde3* RNA was detected with the mouse *Gdpd2* probe (ACDbio, 494171) at 1:50 and the Opal-570 fluorophore (Akoya Biosciences, FP1488001KT) at 1:1500. Slides were imaged on a Zeiss LSM 700 confocal microscope. Cell identity and *Gde3* expression were determined manually.

### Isolation of astrocytes

P5 pups were decapitated, the brain was removed, and placed in dissection media [Hank’s balanced salt solution (Gibco, 14175103) and 1% Penicillin-Streptomycin (PenStrep; Gibco, 15140122)]. Dissected cortices were chopped into small pieces with a razor blade and incubated in dissection media containing 0.25% trypsin (Gibco, 15090046) and 0.05% deoxyribonuclease I (Sigma-Aldrich, DN-25) at 37°C for 15 min. Tissue was then titrated by pipetting 10 times with a P1000 pipette and run through a 70-μm cell strainer to obtain a single-cell solution. Cells were pelleted and resuspended in 10 ml of glia media [MEM (minimum essential media; Gibco 1109508), 10% FBS (fetal bovine serum; Sigma-Aldrich, F4135), and 1% PenStrep] before plating on pretreated 10-cm plates [coated with collagen (1 mg/ml; Advanced Biomatrix, 5005) overnight at 37°C and washed in PBS three times before use]. Plates were incubated in a humidified 37°C incubator with 5% CO_2_. Two days later, cells were mechanically agitated to remove nonastrocytic cells, and the media were removed and replaced. This agitation procedure was repeated daily until the cells were 70 to 80% confluent (typically 7 days) when cells were passaged for experiments.

### Cell culture

#### HEK293T cells

HEK293T cells were grown in HEK media [DMEM (Dulbecco’s modified Eagle’s medium) (Gibco, 31053028), 10% FBS, and 1% PenStrep] in a humidified 37°C incubator with 5% CO_2_. Plates were coated with PEI (polyethylenimine; 25 μg/ml) (Sigma-Aldrich, P3143) for 1 hour and washed with PBS three times before use. When confluent, HEK293T cells were washed in PBS and trypsinized with TrypLE (Gibco, 12563011) for 5 min. Cells were counted on a hemocytometer, and 150,000 cells were added to each well of a 12-well dish and scaled for different-sized plates.

#### Primary astrocytes

Primary astrocytes were used between passages 1 and 5. When 70 to 80% confluent, astrocytes were washed in PBS and trypsinized with TrypLE for 5 min at 37°C and plated on collagen-coated plates. For experiments involving multiple genotypes (WT and *Gde3* KO), equal numbers of cells were used from littermate pups that were isolated and cultured in parallel.

### Plasmids and cloning

Expression plasmids containing GDE3 or GDE3-ΔN are described in (*13*). They contain a pCAGGs promoter before GDE3 and an internal ribosomal entry site–green fluorescent protein after GDE3. The TurboID sequence was obtained from Addgene (107169) (*30*) and cloned into the GDE3 or GDE3-ΔN vectors using Gibson cloning.

The WAVE2-mCherry plasmid was obtained from Adgene (55161). The WAVE3-mCherry plasmid was generated by cloning WAVE3 from adult mouse hippocampus cDNA using 5′ CAGATCCGCTAGCGCCACCATGCCTTTAGTGAAGAGAAACATCGAAC and 5′ CGGGCCCGCGGTACCCCGCTACCACTTCCGCCGCTGTCAGACCAGTCATTCTCATCAAAC primers and inserted into the WAVE2-mCherry vector via Gibson cloning.

For imaging of GDE3 expression in HEK293T cells, GDE3 was stably expressed using a plasmid that expresses GDE3-P2A-puromycin (backbone provided by S. Gould). GDE3 expression was maintained at stable levels under puromycin selection.

### Transfection

HEK293T cells were transfected the day after plating with Lipofectamine 2000 (Thermo Fisher Scientific, 11668027) according to the manufacturer’s protocol. Briefly, 25 μl of OptiMEM (Gibco 31985070) was mixed with 1.5 μl of Lipofectamine 2000 and incubated at room temperature for 5 min, then mixed with 25 μl of OptiMEM containing 100 ng of DNA and allowed to incubate for 30 min at room temperature. This mixture was applied to HEK293T cells at 40 to 50% confluency in a 12-well dish. The mixture was scaled up as appropriate for larger plates. The following day, transfected cells were used for experiments.

Primary astrocytes were transfected when cells were 80 to 90% confluent with Lipofectamine LTX (Thermo Fisher Scientific, 15338030) according to the manufacturer’s protocol. Briefly, 25 μl of OptiMEM was mixed with 1.5 μl of Lipofectamine LTX and incubated at room temperature for 5 min. This was then mixed with 25 μl of OptiMEM containing 1 μg of DNA of each plasmid and 1 μl of Lipofectamine PLUS reagent and allowed to incubate for 30 min at room temperature. This mixture was applied to astrocytes in a 12-well dish. This mixture was scaled up as appropriate for larger plates. After 3 hours, cells were washed with warm PBS twice and placed in fresh glia media.

### siRNA knockdown

Primary astrocytes were transfected with siRNAs when cells were 80 to 90% confluent with RNAiMAX (Thermo Fisher Scientific, 13778030) according to the manufacturer’s protocol. siRNAs were purchased from Santa Cruz Biotechnology (Scrambled: sc-36869, WAVE2: sc-36834, WAVE3: sc-43499). Briefly, 166 μl of OptiMEM containing 33.3 pmol of siRNA was mixed with 166 μl of OptiMEM containing 6.7 μl of RNAiMAX and incubated for 5 min. Then, 330 μl of this mixture was applied to each well of a six-well dish. Twenty-four hours later, cells were washed twice in PBS, and DMEM without serum was added to the cells for EV collection (see the “EV isolation by centrifugation” section below).

### EV isolation by centrifugation

We have submitted all EV protocols to the EV-TRACK knowledgebase (EV-TRACK ID: EV230374) (*54*). HEK293T cells or primary astrocytes in a 12-well plate were washed twice in PBS before the addition of 1 ml of serum-free media (DMEM and 1% PenStrep). Twenty-four hours later, media were removed from cells and transferred to a tube containing 10 μl of protease inhibitors and placed on ice. Cells were washed once with PBS, and then lysed directly in 1 ml of GLB for 20 min with shaking. The lysed samples were then sonicated briefly, and insoluble material was removed by centrifugation.

One milliliter of media containing EVs was spun at 3000*g* for 10 min at 4°C to remove dead cells and apoptotic bodies, transferred to a new tube, and spun at 3000*g* again for 10 min at 4°C to purify total EVs. Nine hundred microliters of EVs was then spun at 12,000*g* for 1 hour at 4°C to pellet large vesicles. For isolation of small vesicles, the supernatant for the 12,000*g* spin was transferred to ultracentrifuge tubes (Beckman, 362305) and spun at 100,000*g* for 70 min at 4°C (Beckman Optima TLX Ultracentrifuge with TLA100.4 rotor). The supernatant was discarded. The 12,000 and 100,000 pellets were resuspended in 90 μl of GLB for Western blots or 900 μl of cold PBS for NTA analysis. This isolation was scaled up for larger volumes of media as necessary.

The CD63 and CD81 antibodies used for Western blots do not work when BME is present in the GLB. Therefore, BME was excluded from the GLB when a sample was going to be blotted for CD63 or CD81. BME was added into aliquots of these samples for Western blotting with other antibodies.

### EV isolation by SEC

#### HEK293T cells

HEK293T cells were cultured and transfected as described above. Total EVs were collected as above with two successive 3000*g* spins. Size exclusion columns (Izon, SP5) were used according to the manufacturer’s protocols. Briefly, 1-ml qEVoriginal/35-nm columns were washed with 10 ml of PBS. Five hundred microliters of total EVs was added and allowed to flow through, followed by 2 ml of PBS; the first 2.5-ml volume to flow through the column was collected as the void. Subsequently, 500 μl of PBS was added to the column, allowed to flow through, and collected as fraction 1. This was repeated a total of five times. Last, 1 ml of PBS was added and collected as the protein fraction. All fractions were mixed with 4× GLB and frozen for subsequent Western blotting.

#### Astrocytes

WT or *Gde3* KO astrocytes were cultured as described above until 80 to 90% confluent. The cells were washed twice with PBS before the addition of neuronal maintenance media (Neurobasal Media, 2 mM glutamine, 5% B27^+^, and 1% PenStrep). For pharmacology experiments, 0.1% DMSO or 100 μM CK666 (Sigma-Aldrich, SML0006) (in DMSO) was added to the media. Twenty-four hours later, the medium was collected and centrifuged at 3000*g* twice to isolate total EVs. Size exclusion columns (Izon, SP7) were used according to the manufacturer’s protocols. For mass spectrometry experiments, PBS was used as the buffer, and, for electrophysiology experiments, minimal holding artificial cerebrospinal fluid (mhACSF) (92 mM NaCl, 2.5 mM KCl, 1.25 mM NaH_2_PO_4_, 20 mM Hepes, and 25 mM glucose) was used. Briefly, 10-ml qEVoriginal/35 nm columns were equilibrated with 250 ml of filtered buffer; then, 10 ml of total EVs was added and allowed to flow through, followed by 10 ml of buffer. The next 15-ml volume to flow through was collected as EVs. One milliliter was used for quantification by NTA (see below), while the remaining was frozen at −80°C for later use. Columns were reused up to five times and washed with 150 ml of buffer, 5 ml of 0.5 M NaOH, and another 150 ml of buffer between uses.

### Nanoparticle tracking analysis

EV concentration and size from HEK293T cells or astrocytes were quantified using a ZetaView Nanoparticle Tracker (Particle Metrix) and corresponding ZetaView software. A 100-nm nanosphere size standard was used to calibrate the instrument before readings. For each sample, ~1 ml of EVs in PBS or mhACSF was injected into the sample-carrier cell. EVs were counted at a sensitivity of 80. HEK293T EVs were measured at one position with five cycles of reading per position. Astrocytes EVs were measured at 11 positions with one cycle of reading per position. If more than 1 × 10^8^ EVs were counted, then samples were diluted and quantified again. The machine was washed after every sample with 5 ml of PBS or mhACSF.

### EV analysis by ExoView

HEK293T cells were cultured and transfected with FugeneHD (*13*). The following day, cells were washed twice in PBS, and the media were replaced with serum-free DMEM and incubated overnight. Total EVs were collected as above with two successive 3000*g* spins. The ExoView assay was performed according to the manufacturer’s protocol (NanoView Biosciences). Briefly, 35 μl of media containing EVs was added directly onto the surface of ExoView Tetraspanin chips (NanoView Biosciences EV-TETRA-C) overnight. Chips were washed three times; then, fluorescently tagged α-CD63, α-CD81, and α-CD9 antibodies were added to the chips and incubated for 1 hour at room temperature. Chips were then washed three more times, dried, and scanned on an ExoView R100 machine (NanoView Biosciences). Data were analyzed using a custom R code (available upon request). Fluorescent signals corresponding to a determined size were quantified. The background threshold was set at three SDs above the mean fluorescence of the immunoglobulin G capture antibody spot for each fluorescent channel.

### Brain slice preparation for electrophysiology

Male and female mice were used for acute-slice electrophysiological experiments (see table S1 for sample sizes and sexes used for each experiment). All experiments were performed with WT and *Gde3* KO mice at ages P12 to P15. Acute 300-μm thick coronal slices containing hippocampal sections were prepared on the basis of published protocols (*55*). Briefly, slices were cut in a chilled dissection solution [92 mM *N*-methyl-d-glucamine, 2.5 mM KCl, 1.25 mM NaH_2_PO_4_, 30 mM NaHCO_3_, 20 mM Hepes, 25 mM glucose, 2 mM thiourea, 5 mM sodium ascorbate, 3 mM sodium pyruvate, 0.5 mM CaCl_2_·2H_2_O, and 10 mM MgSO_4_·7H_2_O (pH 7.3 to pH 7.4)]. The solution was continually perfused with 95% O_2_ and 5% CO_2_ before and during the slicing procedure. Forty milliliters of the same solution was used in the initial recovery chamber, where the slices sat for 25 min at 32°C. During this incubation, a 2 M NaCl spike-in solution was added to the dissection solution as follows: 66 μl at 0 and 5 min, 133 μl at 10 min, 266 μl at 15 min, and 533 μl at 20 min to yield a final concentration of 92 mM NaCl. At the conclusion of the 32°C incubation, slices were transferred to 100 ml of a room-temperature hACSF solution [92 mM NaCl, 2.5 mM KCl, 1.25 mM NaH_2_PO_4_, 30 mM NaHCO_3_, 20 mM Hepes, 25 mM glucose, 2 mM thiourea, 5 mM sodium ascorbate, 3 mM sodium pyruvate, 2 mM CaCl_2_·2H_2_O, and 2 mM MgSO_4_·7H_2_O (pH 7.3 to pH 7.4)]. For EV experiments, H_2_O, 2 mM thiourea, 5 mM sodium ascorbate, 3 mM sodium pyruvate, 2 mM CaCl_2_·2H_2_O, and 2 mM MgSO_4_·7H_2_O were added to the mhACSF containing EVs (described in EV isolation for electrophysiology section) so that the final 15-ml holding solution had the same ion content as hACSF with a final EV concentration of 5 × 10^7^ EVs/ml. Slices were incubated for a minimum of 1 hour in the holding solution before proceeding with patch-clamp recordings.

### Electrophysiology

Recordings and analysis were carried out blind to mouse genotype and EV condition. CA1 pyramidal neurons near the surface of the slice were visualized with infrared differential interference contrast optics and patched using borosilicate pipettes (2 to 4 megohms). Whole-cell voltage-clamp recordings were performed using a MultiClamp 700B amplifier (Molecular Devices) and conducted in recirculated extracellular recording ACSF solution [124 mM NaCl, 2.5 mM KCl, 1.25 mM NaH_2_PO_4_, 24 mM NaHCO_3_, 5 mM Hepes, 12.5 mM glucose, 2 mM CaCl_2_·2H_2_O, 2 mM MgSO_4_·7H_2_O, 1 μM tetrodotoxin (Abcam ab120054), and 40 μM bicuculline (Sigma-Aldrich, 14343) (pH 7.3 to pH 7.4)]. Recording ACSF was continually aerated with 95% O_2_ and 5% CO_2_ and recycled. For Bay/MPEP treatments, 10 μM of Bay 36-7620 (Tocris, 2501) and 5 μM MPEP hydrochloride (Tocris, 1212) were added to the recording ACSF, and slices were incubated in the recording ACSF for at least 30 min before recording.

Electrophysiological experiments were performed in a voltage clamp at −60 mV using an internal solution [135 mM CsMeSO_3_, 10 mM CsCl, 10 mM Hepes, 0.2 mM EGTA, 3 mM Na_2_ATP, 0.3 mM Na_3_GTP, and 5 mM QX 314 (pH 7.3 to pH 7.4)]. All data were acquired and analyzed using pCLAMP 10 (Molecular Devices), Wdetecta, and Wscnslct (Huguenard laboratory, Stanford). After patching onto cells, 1 min was allowed to elapse to achieve equilibrium, and then the following 9 min of data was collected for analysis. Access and membrane resistances were monitored throughout, and any cell that had an access resistance over 25 megohms or membrane resistance under 150 megohms at any point was excluded. Event detection parameters were optimized for noise level, and a 5-pA amplitude cutoff was used as a threshold. The automated software initially detected events, and then non-events due to noise were manually excluded upon visual inspection of the traces. Any cell that had fewer than 40 total events was excluded from the analysis.

Representative waveform traces were constructed by averaging all events from four cells (>200 events). Bar graphs depicting mean amplitudes and frequencies were constructed by averaging all events of each cell. Cumulative probability plots were constructed from 40 random events per cell to avoid bias from more active cells.

### Tissue and cell culture staining

Slides were deparaffinized by baking at 60°C for 20 min, incubated in xylene (Thermo Fisher Scientific, X3P) for 5 min (three times), and then rehydrated in successive 100, 95, and 70% ethanol washes (twice each wash). Samples were permeabilized in PBST (PBS with 0.3% Triton X-100) for 5 min (three times). Antigen retrieval was done by incubating slides at 98°C for 20 min in antigen retrieval buffer [10 mM sodium citrate and 0.05% Tween 20 (pH 6.0)] and then cooled on ice. Slides were washed in PBST three more times. Cultured cells on coated coverslips were washed in PBS and fixed in 4% PFA in phosphate buffer for 15 min on ice. Cells then were washed in PBS three times for 5 min. After washing, slides or coverslips were blocked in PBST containing 5% BSA (bovine serum albumin) for 1 hour. Primary antibodies were diluted in 1% BSA in PBST (at the concentrations indicated below) and incubated overnight at 4°C. The next day, slides or coverslips were washed in PBST three times for 5 min. Appropriate secondary antibodies at 1:500 and Hoechst at 2 mM were diluted in PBS and incubated with slides or coverslips for 1 hour at room temperature in the dark. Slides or coverslips were washed in PBST three times for 5 min, and then mounted with ProlongGold (Thermo Fisher Scientific). Primary antibodies were used at the following concentrations: 1:500 for α-S100β (DAKO), 1:500 for α-FLAG-M2 (Millipore), 1:100,000 for α-GFAP (DAKO), 1:50 for α-ALDH1L1 (Abcam), and 1:5000 for α-mCherry (Sigma-Aldrich). Cells on coverslips were imaged on a Zeiss LSM 700 confocal microscope, and tissue slides were imaged on a Keyence BZ-X710 widefield microscope. Astrocytes in tissue sections were counted automatically by the number of S100β^+^ cells/mm^2^ using Fiji (FIJI Is Just ImageJ).

### EV staining on coverslips

HEK293T cells were cultured and transfected as described above. The 12,000 EVs were isolated as described above. Coverslips were coated with poly-L-lysine (0.5 mg/ml; Sigma-Aldrich, P2636) at 37°C overnight, and then coated with laminin (10 mg/ml; Sigma-Aldrich, L2020) at 37°C overnight; after coating, coverslips were washed three times in PBS. EVs were diluted 1:50 and 50 μl of EV was placed on the coverslips and allowed to adhere for 1 hour at room temperature. EVs were then fixed to the coverslips with 4% PFA for 15 min at room temperature, and then washed three times in PBS. EVs were then stained as described above with primary antibodies α–annexin A1 (Abcam) at 1:1000 and α-FLAG-M2 (Sigma-Aldrich) at 1:1000.

### Actin polymerization assay

The assay was performed according to the manufacturer’s protocol (Cytoskeleton Inc., BK037). Briefly, astrocytes from a six-well plate at 60 to 70% confluency were lysed in 200 μl of warm LAS02 buffer using a 25G needle. After a 10-min incubation at 37°C, the lysate was spun at 100,000*g* for 1 hour at 37°C. The supernatant was saved as the G-actin fraction, and the pellet was solubilized in an F-actin depolymerizing buffer for 1 hour on ice and saved as the F-actin fraction. Equal volumes of F-actin and G-actin fractions were analyzed by Western blot with the α-actin antibody (1:1000) provided in the kit. Experiments were done in technical duplicate; any samples with an F/G ratio < 0.01 were excluded.

### Mass spectrometry of EVs

WT and *Gde3* KO astrocytes were cultured, and EVs were isolated by SEC as described above. Proteins from 13 ml of EVs were isolated by trichloroacetic acid (TCA); briefly, TCA was added to 20%, vortexed, incubated on ice for 30 min, and spun at 20,000*g* for 30 min at 4°C. The supernatant was removed, and the pellets were washed in 10% TCA, followed by two acetone washes. Pellets were air-dried and stored at −80°C until analysis.

Mass spectrometry analysis was performed by the Taplin Mass Spectrometry Facility (Harvard). Beads were washed at least five times with 100 μl of 50 mM ammonium bicarbonate, then 5 μl (200 ng/μl) of modified sequencing-grade trypsin (Promega, Madison, WI) was spiked in, and the samples were placed in a 37°C room overnight. The samples were then centrifuged or placed on a magnetic plate if magnetic beads were used, and the liquid was removed. The extracts were then dried in a SpeedVac (~1 hour). Samples were then resuspended in 50 μl of high-performance liquid chromatography (HPLC) solvent A [2.5% acetonitrile (ACN) and 0.1% formic acid] and desalted by a STAGE Tip (*56*). On the day of analysis, the samples were reconstituted in 10 μl of HPLC solvent A. A nanoscale reverse-phase HPLC capillary column was created by packing 2.6-μm C18 spherical silica beads into a fused silica capillary (100-μm inner diameter × ~30-cm length) with a flame-drawn tip (*57*). After equilibrating the column, each sample was loaded via a Famos autosampler (LC Packings, San Francisco, CA) onto the column. A gradient was formed, and peptides were eluted with increasing concentrations of solvent B (97.5% ACN and 0.1% formic acid). As peptides eluted, they were subjected to electrospray ionization and then entered into an LTQ Orbitrap Velos Elite ion-trap mass spectrometer (Thermo Fisher Scientific, Waltham, MA). Peptides were detected, isolated, and fragmented to produce a tandem mass spectrum of specific fragment ions for each peptide. Peptide sequences (and hence protein identity) were determined by matching protein databases with the acquired fragmentation pattern by the software program, Sequest (Thermo Fisher Scientific, Waltham, MA) (*58*). All databases include a reversed version of all the sequences, and the data were filtered to between a 1 and 2% peptide FDR. Peptides with only one read across all six samples were removed from further analysis. Peptide reads were averaged for WT and *Gde3* KO samples. Proteins with a relative difference between WT and *Gde3* KO of greater than 2× were considered different. See table S2.

### Biotin labeling and mass spectrometry

HEK293T cells were plated on PEI-coated 10-cm dishes and transfected with GDE3-TurboID or GDE3-ΔN-TurboID as described above. The next day, fresh media containing 50 μM biotin (Sigma-Aldrich, B45010) were added to cells and incubated for 3 hours. After biotinylation, cells were washed in PBS, lysed in RIPA buffer containing protease inhibitors, sonicated, and centrifuged at 20,000*g* to remove insoluble material. Protein was quantified by BCA Protein Assay Kit; 1.1 mg of protein was added to 50 μl of streptavidin beads, and the beads were rotated overnight at 4°C.

Subsequently, beads were washed for 5 min in 50 mM triethylammonium bicarbonate (TEAB) (Sigma-Aldrich, T7408) four times and were resuspended in a buffer containing 8 M urea, 50 mM TEAB, 40 mM chloroacetamide, and 10 mm tris (2-carboxyethyl) phosphine hydrochloride followed by rotating at room temperature for 1 hour. Urea was diluted to 2 M using TEAB, and sequencing-grade trypsin (10 μg/ml; Promega V5113) was added and rotated overnight at room temperature. Beads were spun down, and 1% trifluoroacetic acid (TFA) was added to soluble peptides. Peptides were loaded into a C18 Stage Tip, washed three times with 0.1% TFA, eluted with 40% ACN and 0.1% TFA, and then dried in a SpeedVac.

The prepared peptides were analyzed on an Orbitrap Fusion Lumos Tribrid Mass Spectrometer coupled with the UltiMate 3000 RSLCnano liquid chromatography system (Thermo Fisher Scientific). The peptides were loaded on Acclaim PepMap100 Nano-Trap Column (100 μm by 2 cm; Thermo Fisher Scientific) and resolved on an EASY-Spray column (50 cm–by–75 μm ID; Thermo Fisher Scientific) using a linear gradient of 10 to 35% solvent B (0.1% formic acid in 95% ACN) at 300 nl/min flow rate over 95 min. MaxQuant (v1.5.5.1) software was used for the identification and quantification of proteins (*59*). The mass spectra were searched against the human UniProt database (released in May 2018 with 73,131 entries), including common contaminant proteins. The used database search parameters are as follows: trypsin as a proteolytic enzyme with up to two missed cleavages, first search peptide mass error tolerance of 20 parts per million (ppm), the main search peptide mass error tolerance of 4 ppm, fragment mass error tolerance of 20 ppm, carbamidomethylation of cysteine (+57.02146 Da) as a fixed modification, and oxidation of methionine (+15.99492 Da) and protein acetyl (+ 42.01056 Da) on N terminus as dynamic modifications. Peptides and proteins were filtered at a 1% FDR (*60*). Proteins with a *q* value < 0.05 and proteins with a *q* value < 0.1 with a fold change >2 were considered significant and used for subsequent analysis; see table S3. GO term analysis was done using the GO database (DOI: 10.5281/zenodo.5725227, released 01 November 2020). The interaction map was done with the STRING database (version 11.5) of interactions of medium confidence or higher or high strength.

### Electron microscopy of EVs

EVs (12,000 and 100,000) were collected from transfected HEK293T cells or primary astrocytes as described above. Eight microliters of EV samples was adsorbed to glow discharged (EMS GloQube) ultra-thin (UL) carbon–coated 400 mesh copper grids (Electron Microscopy Sciences CF400-Cu-UL) by flotation for 2 min. Grids were rinsed for 1 min in TBS three times and negatively stained in two consecutive drops of 1% uranyl acetate (aqueous), and then quickly aspirated. Grids were imaged on a Hitachi 7600 TEM operating at 80 kV with an AMT XR80 CCD (8 megapixels).

### Electron microscopy of synapses

Mice were perfused for 1 min with room-temperature PBS followed by 21 ml of chilled 2% PFA and 2% glutaraldehyde in a 0.1 M sodium cacodylate buffer over the course of 7 min. The bodies were kept at 4°C for 2 hours before the brain was removed and shaken overnight in 5 ml of the 2% PFA and 2% glutaraldehyde in a 0.1 M sodium cacodylate buffer. The next day, hippocampal regions were dissected out and postfixed with 2% osmium. Samples were then dehydrated with ethanol before infiltration with propylene oxide and EPON resin. After embedding in resin, the hippocampus was trimmed to the CA1 region and sectioned at 70 nm. Sections were stained with UranyLess and Lead Citrate (Electron Microscopy Sciences). Images were acquired on a Thermo-Fisher Talos L120C G2 Electron microscope at 120 kV and imaged with a 16-megapixel complementary metal-oxide semiconductor camera.

### Western blot

Samples in GLB were boiled for 5 min. BME was added to a final concentration of 0.1% to aliquots of samples that were not used for the detection of CD63 or CD81. Fifteen microliters of samples and 5 μl of protein ladder were loaded on a tris-glycine gel [resolving: 7.5 to 10% acrylamide, 0.375 M tris, and 0.1% SDS (pH 8.8); stacking: 5% acrylamide, 0.125 M tris, and 0.1% SDS (pH 6.8)] and run with tris-glycine buffer (25 mM tris, 192 mM glycine, and 1% SDS) at 120 V for 90 to 120 min. Gels (7.5%) were used for FLAG and calnexin; 10% gels were used for all other antibodies. Gels were transferred to a methanol-soaked PVDF (polyvinylidene fluoride) (Millipore, IPVH00010) membrane for 70 min at 100 V in transfer buffer (25 mM tris, 192 mM glycine, and 10% methanol). Membranes were blocked in 5% nonfat milk in TBST [200 mM NaCl, 50 mM tris, and 0.3% Tween 20 (pH 7.4)] for 1 hour. Membranes were incubated in primary antibodies (at the concentrations indicated below) in 5% nonfat milk in TBST overnight at 4°C with gentle shaking. The following day, membranes were washed three times for 10 min in TBST. Appropriate secondary antibodies (Kindle Biosciences, R1005 or R1006) at 1:10,000 or 1:1000 in 5% nonfat milk in TBST were applied to membranes for 1 hour at room temperature with gentle shaking. Membranes were then washed three to six times for 10 min in TBST. ECL (enhanced chemiluminescence) (Kindle Biosciences, R1002) substrate was applied to membranes for 4 min, and membranes were immediately imaged using the KwikQuant Imager (Kindle Biosciences). If necessary, membranes were stripped of secondary antibodies with hydrogen peroxide for 20 min at room temperature or 1 mM sodium azide overnight at 4°C. After stripping, membranes were washed five times in TBST, and a new primary antibody was applied. The primary antibodies are the following: α-actin (1:10,000), α–annexin A1 (1:10,000), α-calnexin (1:1000), α-CD63 (1:1000), α-CD81 (1:1000), α-FLAG (1:10,000), α-GAPDH (1:1000), α-GFAP (1:1000), α-GLAST (1:200), α-GLT1 (1:2000), α-MCT4 (1:5000), α-WAVE2 (1:1000), and α-WAVE3 (1:1000). Western blots were quantified on Fiji. The release of proteins in EVs was quantified as the amount of protein in the 12,000 or 100,000 fractions divided by the amount of protein in the lysate for each sample.

### Statistics

Data were processed in Excel, and all statistical analysis and graphing were done on GraphPad Prism 9.3. Statistical details (sample size, statistical test, and exact *P* values) for each experiment can be found in table S1.

## Notes

View/request a protocol for this paper from *Bio-protocol*.
