# Supplementary material for: An independent regulator of global release pathways in astrocytes generates a subtype of extracellular vesicles required for postsynaptic function
Source: Sci Adv. 2023 Jun 23;9(25):eadg2067. doi: 10.1126/sciadv.adg2067 (PMC10289663; doi:10.1126/sciadv.adg2067)
Supplement: Supplementary file 1 — Legends for tables S1 to S3 Figs. S1 to S8 [file sciadv.adg2067_sm.pdf]

Science Advances
AAAS

Supplementary Materials for

An independent regulator of global release pathways in astrocytes generates a subtype of extracellular vesicles required for postsynaptic function

Reuben Levy-Myers *et al.*

Corresponding author: Shanthini Sockanathan, ssockan1@jhmi.edu

Sci. Adv. **9**, eadg2067 (2023)
DOI: 10.1126/sciadv.adg2067

The PDF file includes:

Legends for tables S1 to S3
Figs. S1 to S8

Other Supplementary Material for this manuscript includes the following:

Tables S1 to S3

SUPPLEMENTAL INFORMATION

Table S1: Statistical tests and details.

Table with statistical details for all graphs where statistics were performed. The table includes information on sample size, definition of biological and technical replicates, statistical test, posthoc test (if used), and p-values for each comparison tested.

Table S2: Proteomic analysis of GDE3 EVs by mass spectrometry.

Table with the full list of all peptides detected in EVs prepared from WT and *Gde3* KO astrocytes by mass spectrometry (Fig. 2F). Raw peptide counts from each replicate are shown. Additionally, the change between peptides identified in EVs from WT and *Gde3* KO astrocytes, associated classification, and GO terms for affected proteins are also shown.

Table S3: GDE3 interactome identified by mass spectrometry

Table with the full list of all peptides detected in mass spectrometry analysis of proteins interacting with the GDE3 N-terminal domain (GDE3-ΔN vs GDE3) (Fig. 3A). Effect size and statistical information are shown. The category column indicates which group each protein was included in for subsequent analysis.

Figure S1: Properties of MVs released by GDE3 in HEK393T cells.

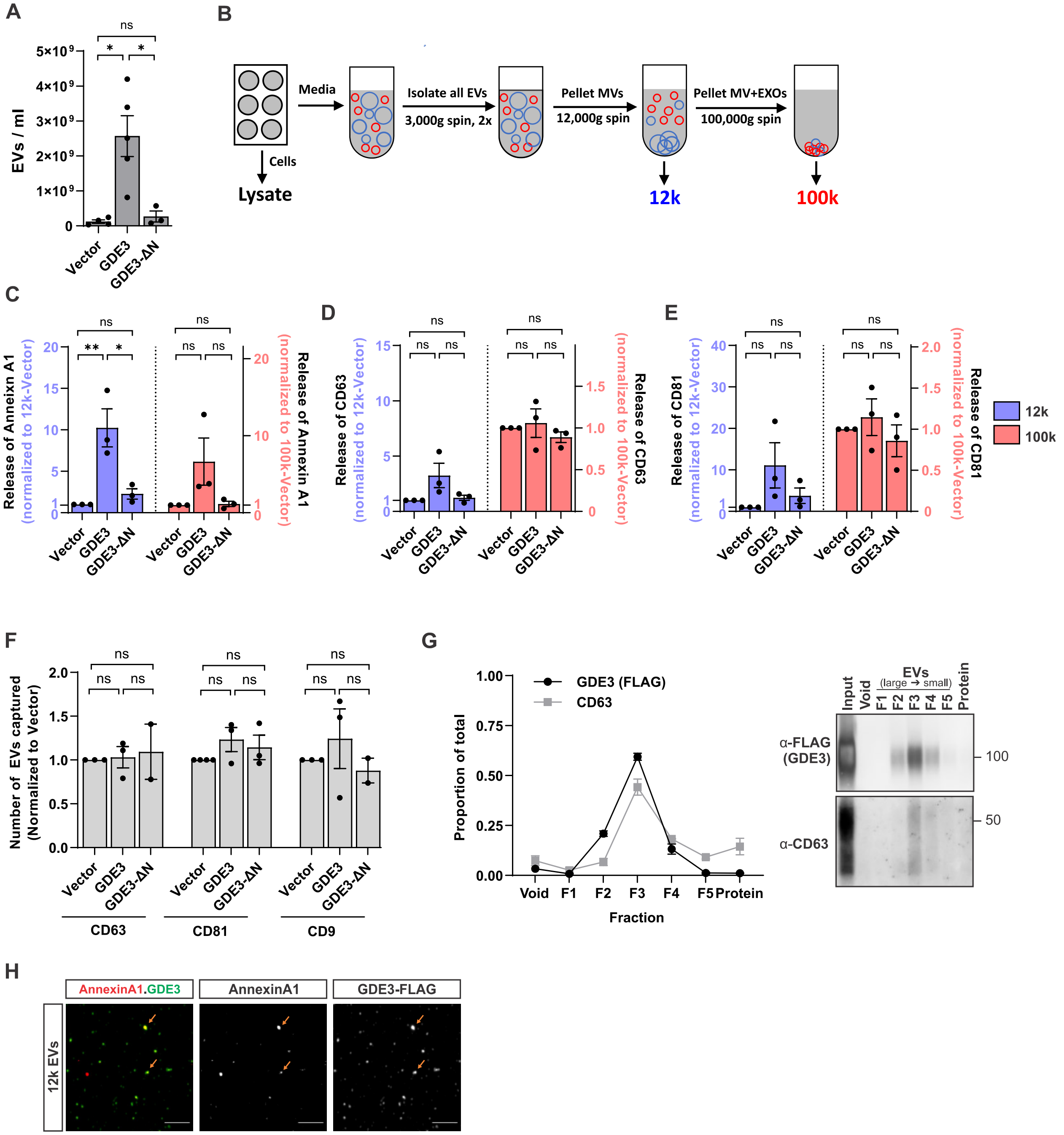

Figure S1: Properties of MVs released by GDE3 in HEK393T cells.

(A) Graph quantifying EV release of transfected HEK293T cells by NTA (see **Figure 1A** for normalized values). (B) Schematic of EV isolation by sequential ultracentrifugation. (C-E) Graphs quantifying the release of AnnexinA1 (C), CD63 (D), and CD81 (E) in 12k and 100k EVs. While

not significant, GDE3 appears to elevate Annexin A1 in 100k EVs and CD63 and CD81 in 12k EVs, likely due to the expression of the latter proteins on the plasma membrane (*21*). (F) Graphs quantifying the number of total EVs containing CD63, CD81, or CD9 from transfected HEK293T cells captured on ExoView chips. (G) Graph and representative Western blot quantifying the proportion of GDE3 (FLAG, Black) and CD63 (gray) in fractions from size exclusion columns (SEC). (H) 12k EVs from HEK293T cells expressing GDE3 stained with FLAG (GDE3) and AnnexinA1. Orange arrows point to EVs with GDE3 and AnnexinA1. The number of EVs containing AnnexinA1 is likely underrepresented due to the difficulty in labeling endogenous AnnexinA1 in single EVs. Scale bar: 5 μm. Data points refer to biological replicates. All graphs are mean $\pm$ SEM. $p > 0.05$ (ns), $p < 0.05$ (*), $p < 0.01$ (**). See **Table S1** for statistical details.

Figure S2: Astrocyte culture and astrocytic EV characteristics

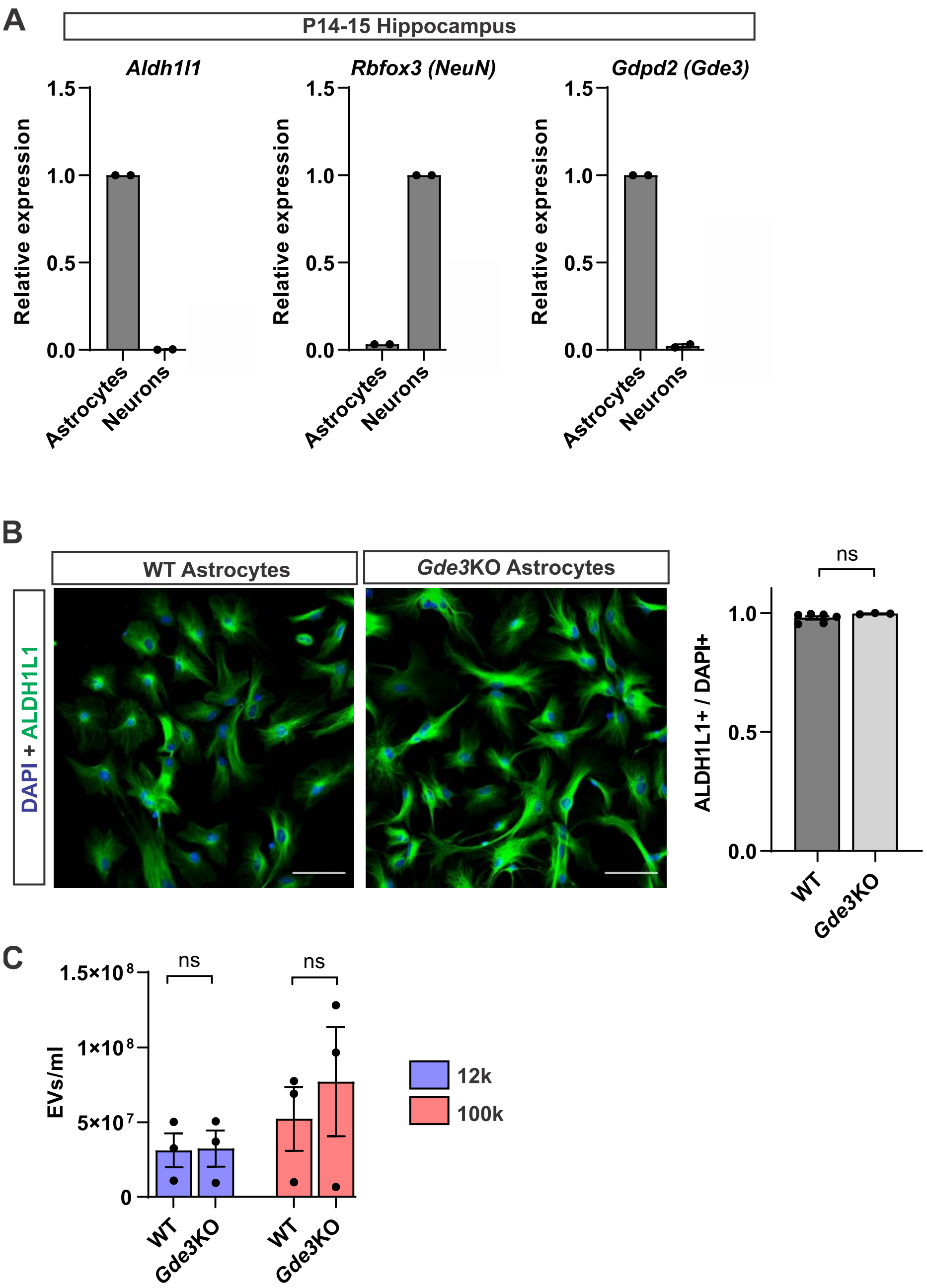

Figure S2: Astrocyte culture and astrocytic EV characteristics

(A) qPCR of cell populations isolated by magnetic cell sorting. Relative expression of astrocyte marker *Aldh1l1*, neuron marker *Rbfox3* (NeuN), and *Gde3* (*Gdpd2*) to *Gapdh*. Values are normalized to cell type. Marker enrichment in the appropriate cell population confirms effective separation by cell type. *Gde3* is highly enriched in astrocytes. (B) Representative image and

quantification of purity of cultured WT and *Gde3* KO astrocytes based on ALDH1L1 staining. Scale bar: 50 µm. (C) Graph quantifying the concentration of EVs prepared from WT and *Gde3* KO astrocytes using NTA. Data points refer to biological replicates. All graphs are mean ± SEM. $p > 0.05$ (ns). See **Table S1** for statistical details.

Figure S3: Identification and reduction of the WRC.

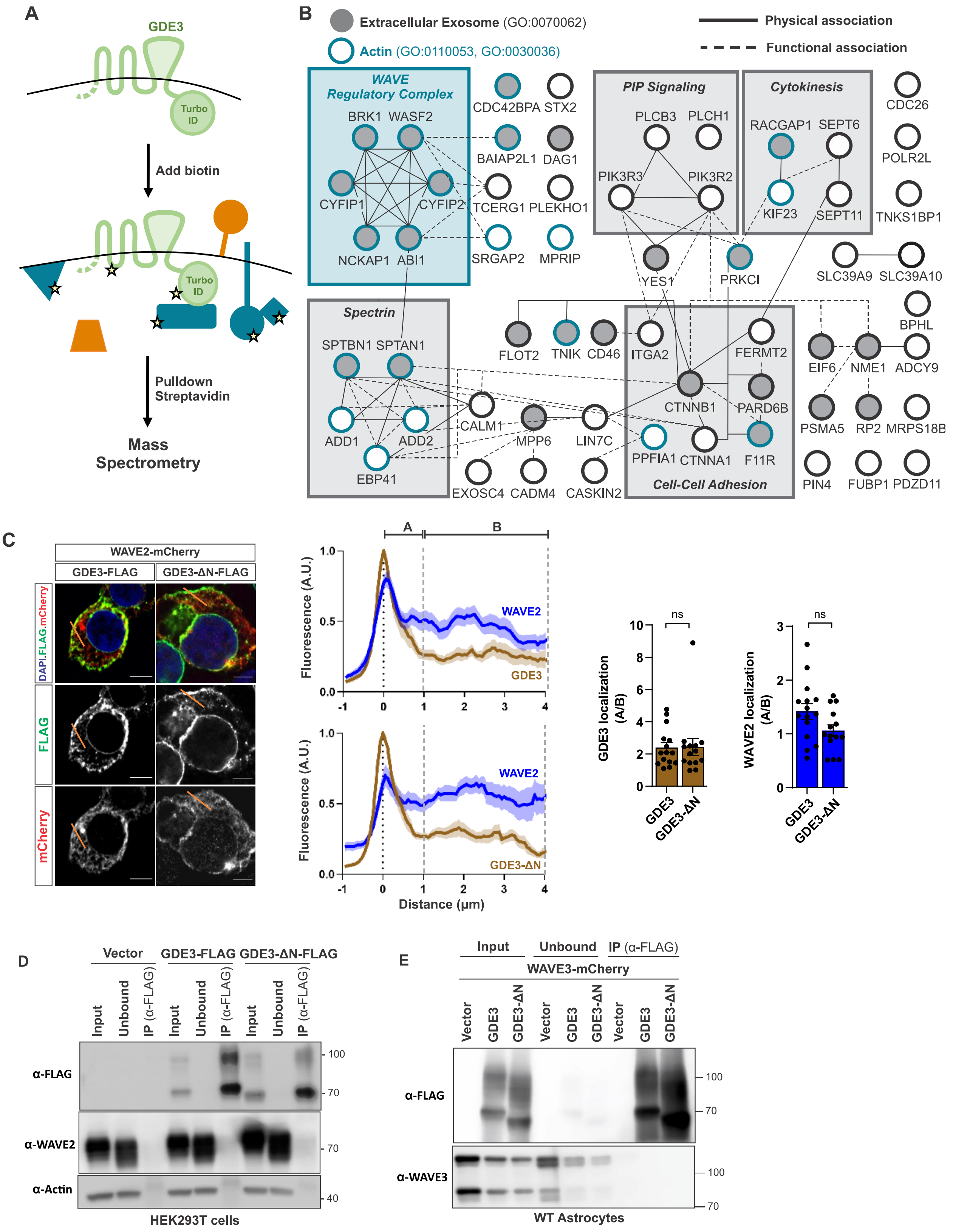

Figure S3: Identification and reduction of the WRC.

(A) Schematic of proximity TurboID screen. (B) Interaction map of proteins with increased proximity to GDE3 (Teal dots in Figure 3A). Lines connecting proteins are based on the STRING database of physical and functional interaction. (C) Representative images of HEK293T cells expressing WAVE2-mCherry and GDE3 or GDE3-ΔN. Scale bar: 10 μm. Fluorescence intensity was quantified along orange lines and graphed. Plots show the average intensity of WAVE2, GDE3 or GDE3-ΔN along the distance of the line; A: surface region (0-1μm); B: Intracellular region (1- 5μm). Bar graphs show the intensity of GDE3 (brown) and WAVE2 (blue) intensities between surface (0-1 μm) and intracellular (1-4 μm) compartments of each cell. (D) Coimmunoprecipitations from HEK293T cells overexpressing empty vector, GDE3, or GDE3-ΔN. Lysates were immunoprecipitated (IP) with anti-FLAG antibodies to pull down GDE3 and GDE3-ΔN. Endogenous WAVE2 was measured by Western blot but not detected in the IP fractions. (E) Coimmunoprecipitations from primary WT astrocytes overexpressing WAVE3-mCherry with empty vector, GDE3, or GDE3-ΔN. Lysates were immunoprecipitated (IP) with anti-FLAG antibodies to pull down GDE3 and GDE3-ΔN. WAVE3-mCherry was measured by Western blot but not detected in the IP fractions. All graphs are mean ± SEM, Data points refer to individual cells, $p > 0.05$ (ns). See **Table S1** for statistical details.

Figure S4: Knockdown of WAVE2 and WAVE3 in astrocytes.

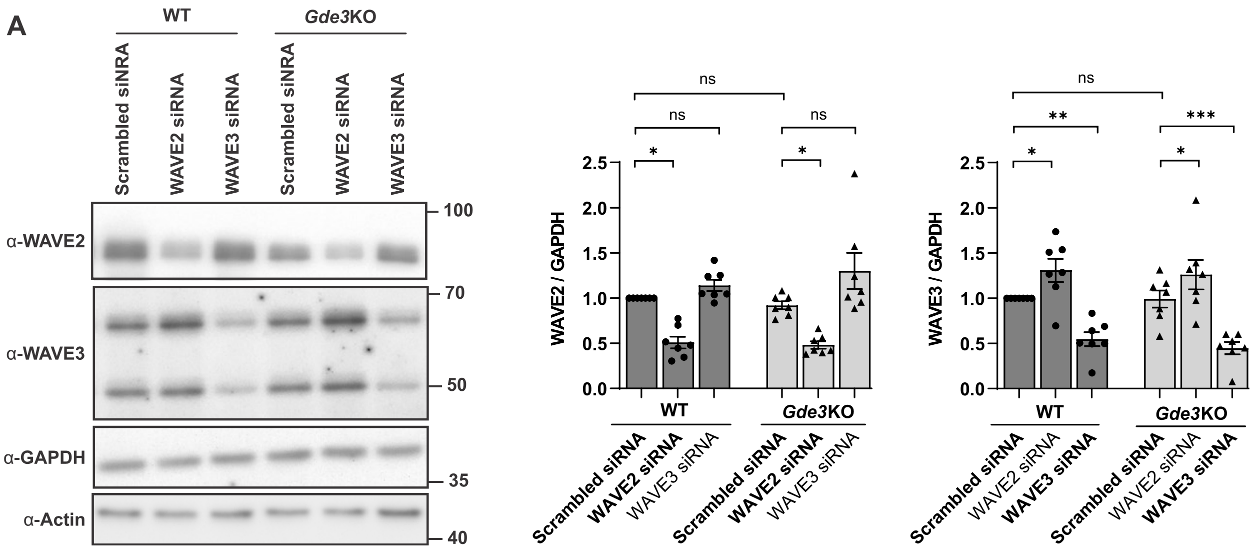

Figure S4: Knockdown of WAVE2 and WAVE3 in astrocytes.

(A) Representative Western blots and quantification of knockdown efficiency of WAVE2 and WAVE3 siRNAs compared to scrambled siRNA. The 65 kDa and 50 kDa bands of WAVE3 were quantified together. Data points refer to biological replicates. All graphs are mean ± SEM. $p > 0.05$ (ns), $p < 0.05$ (*), $p < 0.01$ (**), $p < 0.001$ (***). See **Table S1** for statistical details.

Figure S5: *Gde3* KO astrocytes appear grossly equivalent to WT astrocytes.

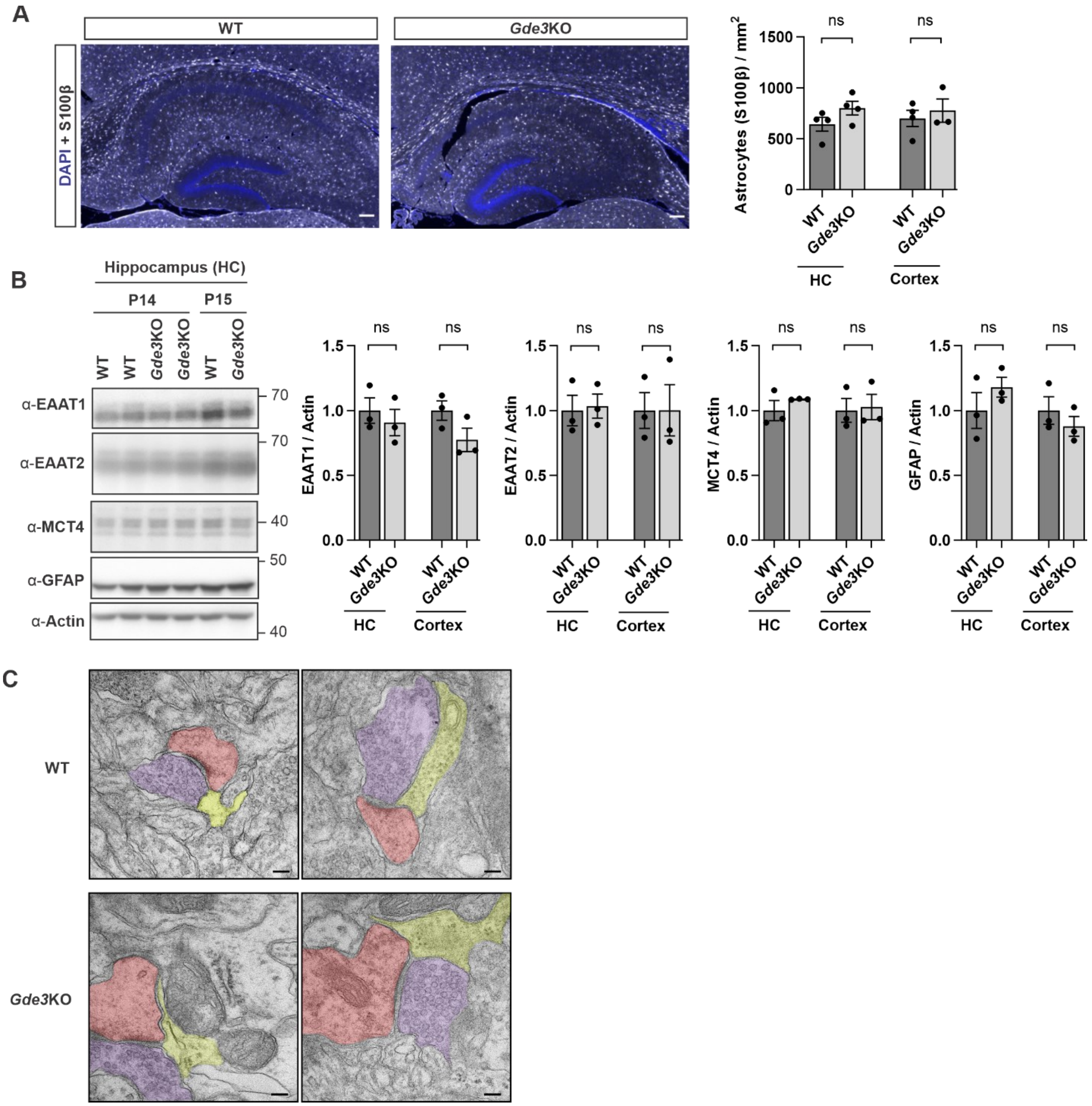

Figure S5: *Gde3* KO astrocytes appear grossly equivalent to WT astrocytes.

(A) Representative images of immunohistochemical staining for the astrocyte marker S100β (white) and DAPI (blue) in the hippocampus of WT and *Gde3* KO P14-15 pups. Scale bar: 100 µm. Right: Graph quantifying the number of S100β-positive cells/mm^2 in the hippocampus (HC) and cortex. (B) Representative western blots for astrocyte-expressed proteins in hippocampal

protein extracts and graphs quantifying expression from western blots of hippocampal (HC) and cortical protein extracts prepared from WT and *Gde3* KO P14-15 pups. (C) Transmission electron micrograph images of synapses in the CA1 region of WT and *Gde3* KO P12-15 mice. Presynaptic neurons are colored in purple, postsynaptic neurons are colored in pink, and astrocytes adjacent to synapses are colored in yellow. Scale bar: 100nm. Data points refer to biological replicates. All bar graphs are mean $\pm$ SEM. $p > 0.05$ (ns). See **Table S1** for statistical details.

Figure S6: Electrophysiological properties are equivalent between WT and *Gde3* KOs.

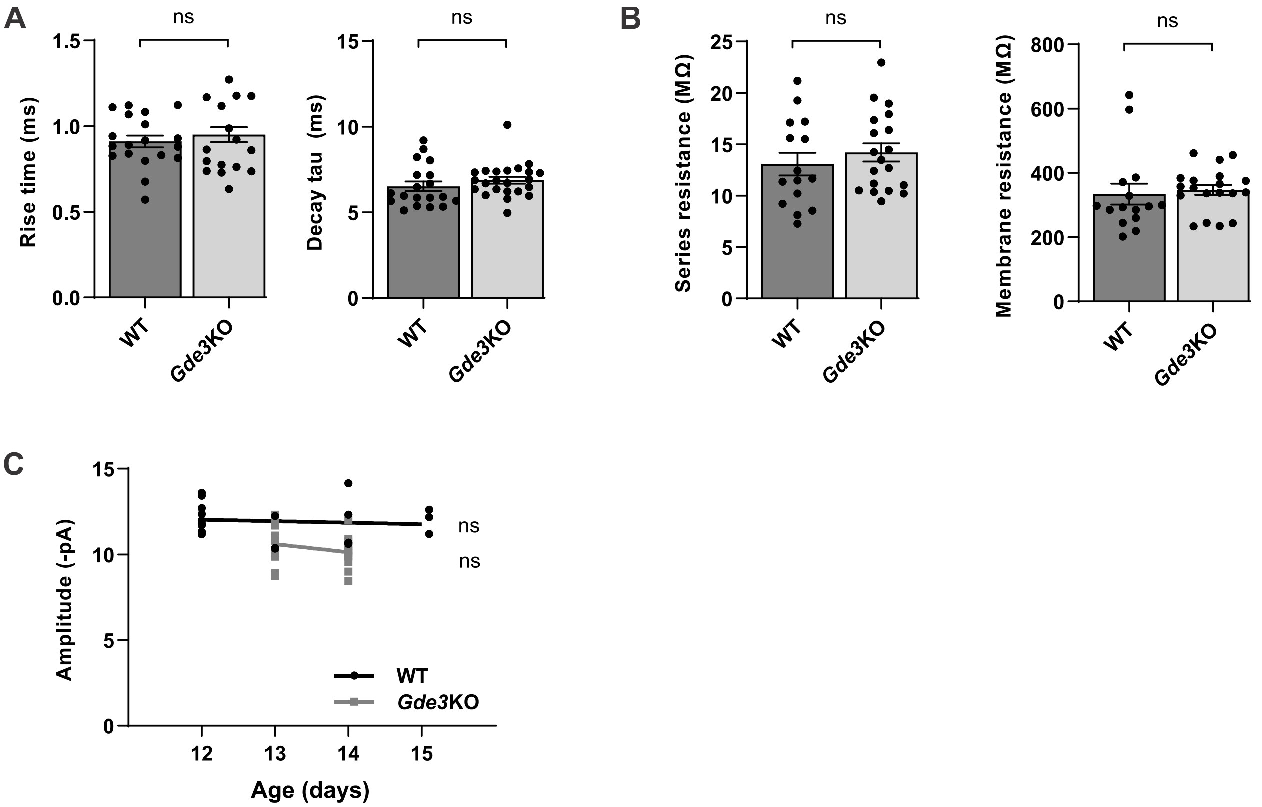

Figure S6: Electrophysiological properties are equivalent between WT and *Gde3* KOs.

(A-C) Measurements in P12-15 WT and *Gde3* KO CA1 cells. (A) Graphs quantifying rise time and decay tau of mEPSCs. (B) Graphs quantifying series and membrane resistances. (C) Graphs quantifying amplitudes in WT and *Gde3* KO CA1 neurons at different ages. The statistical test indicates there is no relationship between age and amplitude. Data points refer to cells from 6 WT and 8 *Gde3* KO mice. All bar graphs are mean ± SEM. p > 0.05 (ns). See **Table S1** for statistical details.

Figure S7: Loss of *Gde*3 in neurons has no effect on mEPCSs

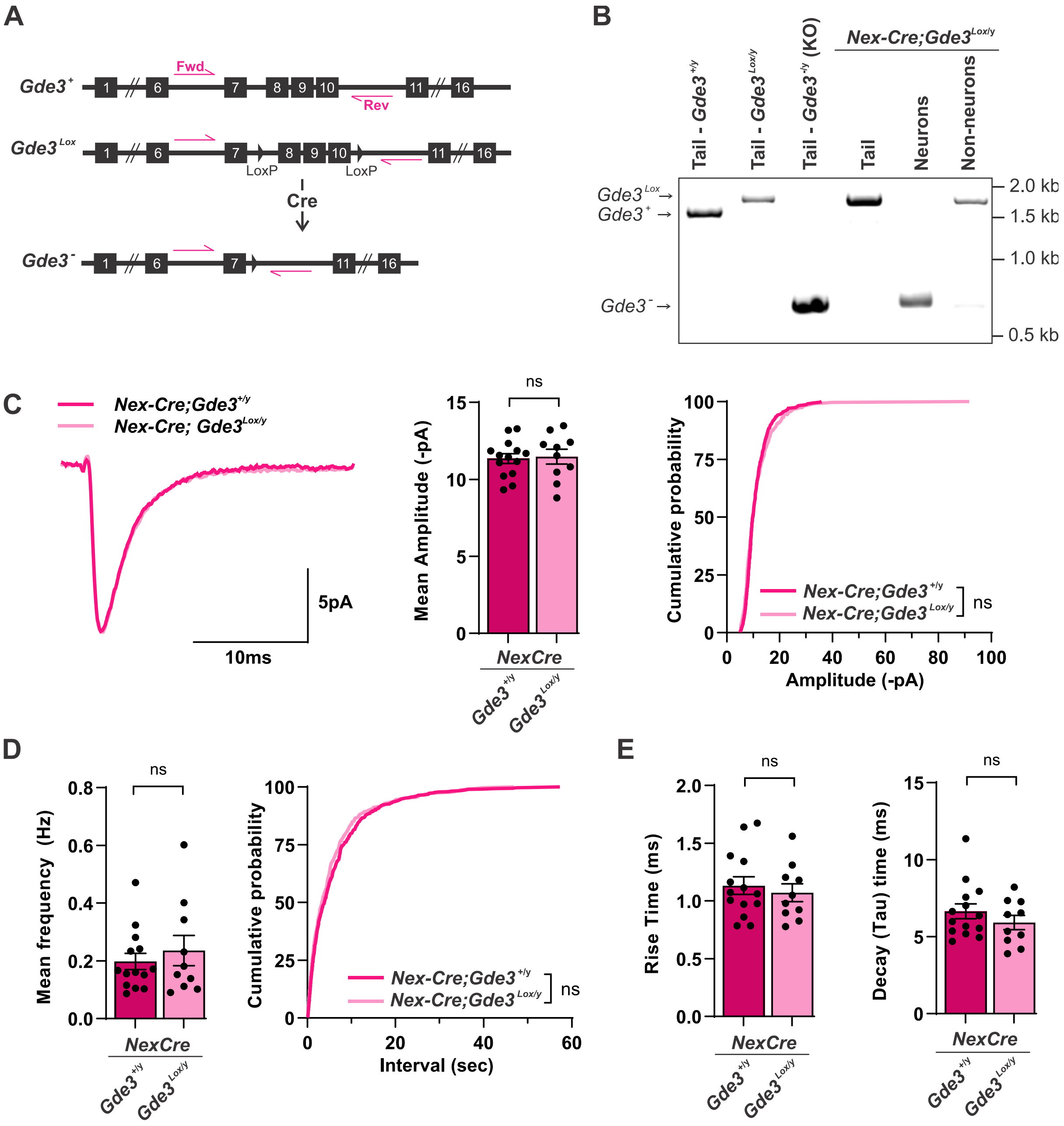

Figure S7: Loss of *Gde*3 in neurons has no effect on mEPCSs

(A) Schematic of WT, conditional *Gde3* Lox allele, and *Gde3* Lox allele after Cre recombination. Primers for PCR are shown. Published studies confirm that this strategy successfully ablates GDE3 protein production (*13*). (B) PCR for the *Gde3* allele from the indicated samples. *Gde3* KO was previously generated using a *CMV-Cre* transgenic mouse (*13*). *Nex-Cre* animals express Cre

recombinase in excitatory neurons (*53*) and thus will remove *Gde3* only from excitatory neurons leaving GDE3 expressed at normal levels in inhibitory neurons and glia. Samples from *Nex-Cre;Gde3*$^{Lox/y}$ pups were collected at embryonic day (E) 13.5. Neurons were isolated from the cortex and hippocampus and separated from other cells using Miltenyi neuronal isolation beads. E13.5 was chosen because this time point is before inhibitory neurons have migrated into the cortex/hippocampus, which would confound the confirmatory PCR on neurons. PCR confirms complete ablation of *Gde3* in E13.5 neurons with *Nex-Cre* but minimal loss of *Gde3* in non-neuronal cells. (C-E) Measurements in P12-15 *Nex-Cre;Gde3*$^{+/y}$ controls and *Nex-Cre;Gde3*$^{Lox/y}$ CA1 cells. (C) Measurements of mEPSC amplitude. Left: superimposed representative averaged traces aligned by rise time. Middle: Graph quantifying the mean amplitude of each cell. Right: cumulative distribution of amplitudes. (D) Measurements of mEPSC frequency. Left: Graph quantifying the mean frequency of each cell. Right: Cumulative distribution of inter-event interval of events. (E) Graphs quantifying rise time and decay tau. Data points refer to cells from 4 *Nex-Cre;Gde3*$^{+/y}$ and *Nex-Cre;Gde3*$^{Lox/y}$ mice. All bar graphs are mean $\pm$ SEM. $p > 0.05$ (ns). See **Table S1** for statistical details.

Figure S8: Characterization of EV treatment on *Gde3* KO neurons.

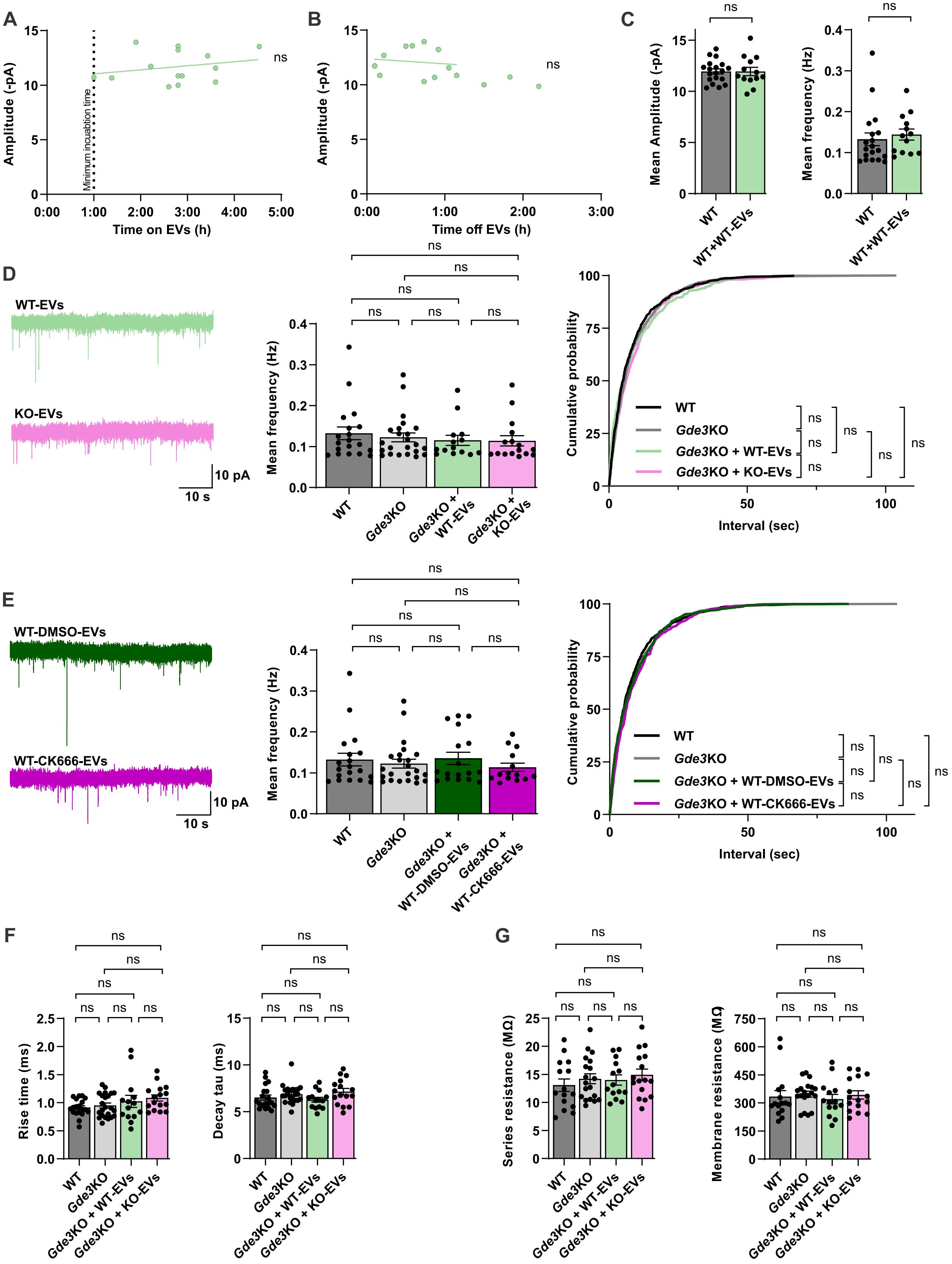

Figure S8: Characterization of EV treatment on *Gde3* KO neurons.

(A) Correlation plots between amplitude and time incubated with WT-EVs for each cell from Figure 5B (green bar). The statistical test used indicates if there is a relationship between time and amplitude (B) Correlations between amplitude and time after removal of WT-EVs for each cell from Figure 5B (green bar). The statistical test used indicates if there is a relationship between time and amplitude. (C) Measurements of mEPSC amplitude and frequency in P12-15 WT slices treated with WT-EVs. (D-G) Measurements of mEPSC frequency in P12-15 *Gde3* KO CA1 cells treated with EVs. (D, E) WT and *Gde3* KO data in D and E are reproduced from Fig. 4B (grey bars, grey and black curves). Left: representative traces over one minute. Middle: Graph quantifying the mean frequency of each cell. Right: Cumulative distribution of inter-event interval of events. (D) Added EVs from WT (WT-EVs, green) and *Gde3* KO (KO-EVs, pink) astrocytes. (E) Added EVs from WT astrocytes treated with DMSO (WT-DMSO-EVs, dark green) and 100 μM CK666 (WT-CK666-EVs, dark pink). (F) Graphs quantifying rise time and decay tau. WT and *Gde3* KO data are reproduced from Fig S6A (grey bars). (G) Graphs quantifying series and membrane resistances. WT and *Gde3* KO data are reproduced from Fig S6B (grey bars). Data points refer to cells analyzed. All bar graphs are mean $\pm$ SEM. $p > 0.05$ (ns). See **Table S1** for statistical details.
